# TFEB induces mitochondrial itaconate synthesis to suppress bacterial growth in macrophages

**DOI:** 10.1038/s42255-022-00605-w

**Published:** 2022-07-21

**Authors:** Ev-Marie Schuster, Maximilian W. Epple, Katharina M. Glaser, Michael Mihlan, Kerstin Lucht, Julia A. Zimmermann, Anna Bremser, Aikaterini Polyzou, Nadine Obier, Nina Cabezas-Wallscheid, Eirini Trompouki, Andrea Ballabio, Jörg Vogel, Joerg M. Buescher, Alexander J. Westermann, Angelika S. Rambold

**Affiliations:** 1 Department of Developmental Immunology, Max Planck Institute of Immunobiology and Epigenetics, Freiburg, Germany; 2 International Max Planck Research School for Immunobiology, Epigenetics and Metabolism (IMPRS-IEM), Freiburg, Germany; 3 Faculty of Biology, University of Freiburg, Freiburg, Germany; 4 Max Planck Institute for Immunobiology and Epigenetics, Freiburg, Germany; 5 Center of Chronic Immunodeficiency, Medical Center University of Freiburg, Freiburg, Germany; 6 Department of Molecular Immunology, Max Planck Institute of Immunobiology and Epigenetics, Freiburg, Germany; 7 IRCAN Institute for Research on Cancer and Aging, INSERM Unité 1081, CNRS UMR 7284, Université Côte d’Azur, Nice, France; 8 Telethon Institute of Genetics and Medicine, Medical Genetics Unit, Department of Medical and Translational Science and SSM School for Advanced Studies, Federico II University, Naples, Italy; 9 Department of Molecular and Human Genetics, Baylor College of Medicine, Jan and Dan Duncan Neurological Research Institute, Texas Children’s Hospital, Houston, TX USA; 10 Helmholtz Institute for RNA-based Infection Research, Helmholtz Centre for Infection Research (HZI), University of Würzburg, Würzburg, Germany; 11 Institute of Molecular Infection Biology, University of Würzburg, Würzburg, Germany; 12 Metabolomics Core Facility, Max Planck Institute of Immunobiology and Epigenetics, Freiburg, Germany

**Keywords:** Phagocytes, Antimicrobial responses, Metabolism

## Abstract

Successful elimination of bacteria in phagocytes occurs in the phago-lysosomal system, but also depends on mitochondrial pathways. Yet, how these two organelle systems communicate is largely unknown. Here we identify the lysosomal biogenesis factor transcription factor EB (TFEB) as regulator for phago-lysosome-mitochondria crosstalk in macrophages. By combining cellular imaging and metabolic profiling, we find that TFEB activation, in response to bacterial stimuli, promotes the transcription of aconitate decarboxylase (Acod1, Irg1) and synthesis of its product itaconate, a mitochondrial metabolite with antimicrobial activity. Activation of the TFEB–Irg1–itaconate signalling axis reduces the survival of the intravacuolar pathogen *Salmonella enterica* serovar Typhimurium. TFEB-driven itaconate is subsequently transferred via the Irg1-Rab32–BLOC3 system into the *Salmonella*-containing vacuole, thereby exposing the pathogen to elevated itaconate levels. By activating itaconate production, TFEB selectively restricts proliferating *Salmonella*, a bacterial subpopulation that normally escapes macrophage control, which contrasts TFEB’s role in autophagy-mediated pathogen degradation. Together, our data define a TFEB-driven metabolic pathway between phago-lysosomes and mitochondria that restrains *Salmonella* Typhimurium burden in macrophages in vitro and in vivo.

## Abstract (web summary)

The key regulator of lysosomal biogenesis, Tfeb, is shown to directly induce Irg1 transcription and mitochondrial itaconate production to restrain bacterial growth in macrophages.

## Main

Lysosomes are critical organelles with degradative and recycling functions and roles in membrane repair, metabolism and signalling^1,2^. In macrophages, the phago-lysosomal compartment has evolved several functions to act as first-line organelle in the defence against foreign invaders; it harbours toll-like receptors and contributes acidic hydrolases and reactive oxygen species-producing membrane complexes to sense, kill and digest sequestered microbes^3–6^. Efficient bacterial detection and elimination also depends on other organelle systems. Mitochondria have emerged as key cellular hubs for the integration of metabolism and phagocyte effector functions^7^. Recent evidence shows a key function of metabolites derived from the mitochondrial tricarboxylic acid (TCA) cycle in modulating inflammatory circuits and microbial control^8–11^. Although it is known that mitochondria can act synergistically and even interact physically with lysosomes in some cell types^12,13^, it remains largely unknown how these two organelle systems communicate or integrate their functions to control bacterial challenges^14–16^.

To identify pathways for potential inter-organellar communication between phago-lysosomes and mitochondria in macrophages, we began our study by modulating TFEB^2,17^. Previous work showed that TFEB is activated upon bacterial uptake via phago-lysosomal calcium release, and benefits bacterial clearance^18^. While TFEB is best known as regulator of lysosomal biogenesis^19^, it is emerging to control pleiotropic processes. The underlying mechanisms for its antimicrobial control remain largely unexplored^18^.

**Fig. 1:**
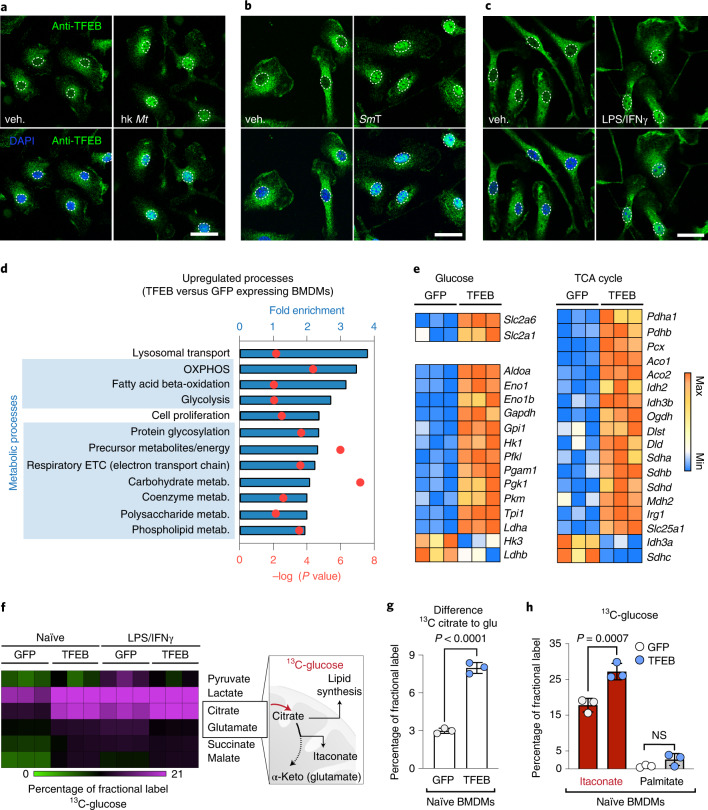
TFEB activation drives metabolic gene expression in macrophages. **a**–**c**, Images of endogenous TFEB visualized by immunofluorescence in BMDMs treated for 30 min with heat-killed *M. tuberculosis* (hk *Mt*, 10 µg ml^−1^) (**a**), living *S*. Typhimurium (*Sm*T, MOI 5) (**b**) or LPS/IFNγ (15 min) (**c**). Dotted lines indicate cell nuclei. Scale bars, 10 µm. Images are representative of *n* = 2 independent experiments. **d**,**e**, Analysis of RNA-seq data. **d**, Top overrepresented biological processes among significantly upregulated genes from RNA-seq analysis of TFEB-GFP-relative to GFP-expressing naïve BMDMs. **e**, RNA-seq analysis of glucose metabolism and TCA-cycle genes that are significantly differentially expressed between TFEB-GFP- and GFP-expressing BMDMs. Data stem from *n* = 1 biological replicate with *n* = 3 technical replicates. **f**–**h**, Metabolic labelling of BMDMs with ^13^C-glucose in the presence or absence of LPS/IFNγ for 6 h measured by GC–MS/MS. **f**, Results of ^13^C fractional label of TCA-cycle metabolites. **g**, Quantification of ^13^C-glucose fractional label lost between citrate and glutamate. **h**, ^13^C-glucose label in itaconate and palmitate in naïve TFEB-GFP- and GFP-expressing BMDMs. **g**,**h**, Bars show mean ± s.d. of *n* = 3 independent biological replicates, *P* values were calculated using unpaired, two-sided Student’s *t*-test (**g**) and one-way analysis of variance (ANOVA) with Tukey’s post hoc test (**h**), NS *P* > 0.05. glu., glutamate, α-keto., alpha-ketoglutarate, veh., vehicle. Source data

**Extended Data Fig. 1:**
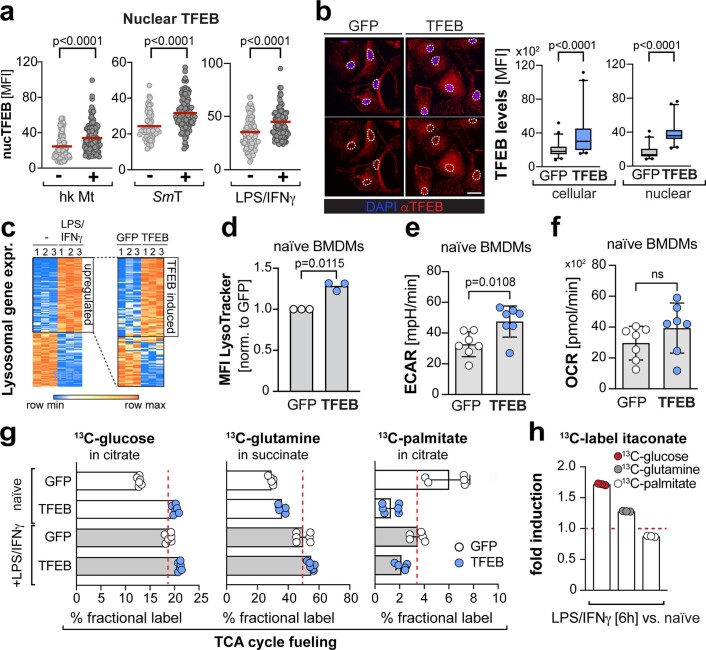
TFEB activation induces the expression of lysosomal and metabolic genes. **a**, Quantification of nuclear TFEB levels from BMDMs treated or not for 30 min with heat-killed *Mycobacterium tuberculosis* (hk *Mt*, 10 µg/mL), living *Salmonella* Typhimurium (*Sm*T, MOI 5), or LPS/IFNγ (15 min), related to Fig. 1a-c. Quantification represents (a) N = 101, 117 (b) N = 56, 115, (c) N = 109, 99 cells examined over *n* = 2 independent experiments. Red lines indicate the mean and p-values were determined using unpaired, two-sided Student’s *t*-test. **b**, Quantification of cellular and nuclear TFEB induction in the TFEB activation mimic and GFP-expressing control cells, quantified from images of cells immune-stained against endogenous TFEB. Scale bar: 10 µm. Box plots (box: 25–75 percentile, middle line: median, whiskers: 5-95 percentile) of N = 52 (GFP), N = 41 (TFEB) cells examined over of *n* = 2 independent experiments. *P* values were calculated using unpaired, two-sided Student’s *t*-test. **c**, Heatmaps of lysosomal genes from RNA-seq analysis in: (left panel) naïve versus 24 h LPS/IFNγ treated, GFP-expressing BMDMs; (right panel) naïve BMDMs expressing TFEB-GFP or GFP. **d**, Quantification of LysoTracker fluorescence in TFEB-GFP- and GFP-expressing BMDMs by flow cytometry. Bars represent mean ± s.d. of *n* = 3 independent experiments. *P*values were calculated using two-tailed one-sample *t*-test. MFI: median fluorescent intensity. **e,f**, Metabolic measurements from TFEB-GFP- or GFP-expressing BMDMs, showing (**e**) extracellular acidification rates (ECAR) and (**f**) oxygen consumption rates (OCR). Bars show mean ± s.d. from *n* = 7 independent biological replicates. Statistics derived from two-sided, unpaired Student’s *t*-tests. n.s. for *P* > 0.05. **g,h**, TCA cycle fueling determined by metabolic labelling of TFEB-GFP- and GFP-expressing BMDMs with ^13^C-glucose, or ^13^C-glutamine, or ^13^C-palmitate in the presence or absence of LPS/IFNγ. Results are based on GC-MS/MS measurements. (**g**) Bar graphs present fractional label of ^13^C-glucose in citrate, ^13^C-glutamine in succinate, and ^13^C-palmitate in citrate. Bars show mean ± s.d. from (left and middle panel) *n* = 6 and (right panel) *n* = 5 (GFP) and *n* = 6 (TFEB) biologically independent samples. (**h**) Itaconate-fueling from ^13^C-labelled glucose, glutamine and palmitate in 6 h LPS/IFNγ-treated BMDMs relative to naïve BMDMs. Bars show mean ± s.d. from *n* = 3 biologically independent samples. Source data

We confirmed that bacterial signals induce the nuclear translocation of TFEB in bone marrow-derived macrophages (BMDMs), including heat-killed *Mycobacterium tuberculosis* (Fig. 1a and Extended Data Fig. 1a), living *Salmonella* Typhimurium (Fig. 1b and Extended Data Fig. 1a), or the combined stimulation with lipopolysaccharides (LPS) and interferon-γ (IFNy) (Fig. 1c and Extended Data Fig. 1a). To identify TFEB-transcriptional targets in BMDMs, we used a retrovirally controlled TFEB-GFP overexpression system, which resulted in a twofold increase of cellular and nuclear TFEB levels relative to green-fluorescent protein- (GFP-) expressing control BMDMs (Extended Data Fig. 1b). Our observed nuclear TFEB increase was comparable to ranges reported for endogenous TFEB upon bacterial stimulation^20^. Using this activation mimic, we performed RNA-sequencing (RNA-seq) and assessed the biological processes enriched in upregulated genes (Fig. 1d,e). As expected, the top hit referred to changes in lysosomal biology (Fig. 1d and Extended Data Fig. 1c), which was reflected in increased total lysosomal mass (Extended Data Fig. 1d). Ten out of the following 11 enriched gene categories were related to metabolic processes (Fig. 1d). In particular, we found genes relevant for cellular and mitochondrial glucose fuelling (Fig. 1e). This included genes encoding glucose transporters (*Slc2a1*, *Slc2a6*) and glycolytic enzymes (*Aldoa*, *Hk1*, *Pfkl*) (Fig. 1e, left panel), transcriptomic signatures that correlated with mildly elevated glycolysis in macrophages (Extended Data Fig. 1e)^21^. Most surprising, however, was the regulation of enzymes that fuel mitochondria with pyruvate (*Pdha*, *Pcx*) and genes that indicated a biosynthetically active TCA cycle (*Slc25a1*, *Irg1*, *Idh3a*) (Fig. 1e, right panel), a key process in activated macrophages^22^.

To analyse the mitochondrial TCA cycle in more detail, we followed the fate of mitochondria-imported carbon from glucose by metabolic labelling of macrophages with ^13^C-glucose. TFEB-activated macrophages incorporated 1.8-times more ^13^C-glucose-derived carbon into citrate than control cells (Fig. 1f and Extended Data Fig. 1g). In contrast, TCA-cycle fuelling with ^13^C-palmitate or ^13^C-glutamine was not or less strongly increased upon TFEB activation (Extended Data Fig. 1g). The elevated ^13^C-glucose label was not retained throughout the full TCA cycle (Fig. 1f,g) to fuel mitochondrial respiration (Extended Data Fig. 1f). Instead, a large portion of ^13^C-label did not reach glutamate (a proxy for α-ketoglutarate) and further downstream metabolites (Fig. 1f,g). Citrate is a pivotal TCA-cycle intermediate and functions as precursor for the de novo synthesis of fatty acids and the TCA-accessory metabolite itaconate^22^ (Fig. 1f). When we followed the fate of glucose-derived carbon downstream of citrate, we found that TFEB activation routed carbon flux primarily into itaconate, an integral metabolite of the pro-inflammatory macrophage response^23^, but not palmitate (Fig. 1h). With this itaconate-fuelling response, TFEB promotes a TCA-cycle state that is normally engaged in macrophages upon bacterial stimulation (Extended Data Fig. 1h)^22^. Thus, our data reveal a previously unappreciated link between TFEB and a biosynthetic TCA-cycle state in macrophages.

**Fig. 2:**
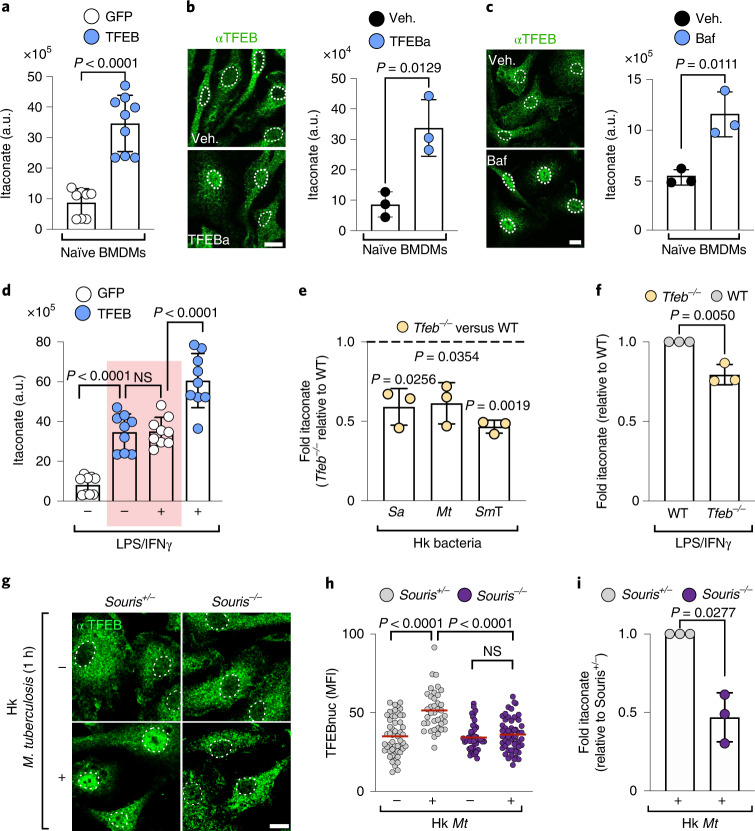
TFEB is a novel regulator of itaconate production. **a**–**c**, Intracellular itaconate levels quantified by LC–MS on the basis of the area under the curve. a.u., arbitrary units. Itaconate was measured in naïve BMDMs (**a**) transduced with the indicated constructs or treated for 24 h with 5 mM TFEBa (**b**) or 100 nM Bafilomycin A1 (Baf) (**c**). Bars show mean ± s.d. of *n* = 9 (**a**) and *n* = 3 (**b**,**c**) independent experiments. *P* values were calculated using unpaired, two-sided Student’s *t*-test. For **b**,**c**, nuclear TFEB translocation was confirmed by immunofluorescence staining. Images are representative for *n* = 2 independent experiments. See Extended Data Fig. 2a for quantification. Scale bars, 10 µm. Dotted lines in the images outline nuclei on the basis of DAPI signals. **d**, Intracellular itaconate levels in naïve and 6 h LPS/IFNγ-treated BMDMs. The red rectangle highlights comparable itaconate levels in naïve TFEB-GFP-expressing and LPS/IFNγ-treated GFP-expressing control BMDMs. Bars show mean ± s.d. of *n* = 9 independent experiments. *P* values were calculated using one-way ANOVA with Tukey’s post hoc. NS with *P* > 0.05. **e**,**f**, Intracellular itaconate levels in TFEB-deficient (*Tfeb*^*−/−*^) and control BMDMs treated with heat-killed (**e**) *S. aureus* (*Sa*, 10^6^ particles per ml), *M. tuberculosis* (*Mt*, 10 µg ml^−1^) and *Salmonella* Typhimurium (*Sm*T, MOI 5) for 10 h or LPS/IFNγ (**f**) for 6 h. Bar graphs represent mean ± s.d., of *n* = 3 independent experiments. *P* values were calculated using two-tailed, one-sample *t*-test. **g**–**i**, Endogenous TFEB activation and quantification of intracellular itaconate levels in *Souris*^*−/−*^ and *Souris*^*+/−*^ BMDMs treated with heat-killed *M. tuberculosis* (10 µg ml^−1^). **g**,**h**, Images depicting TFEB localization (**g**) and quantification of nuclear TFEB levels (**h**) at 1 h post-infection (p.i.) of *n* = 3 independent experiments. Scale bar, 10 µm. Graph shows a mean of *n* = 52, 38 (*Souris*^*+/−*^ with, without hk *Mt*) and *n* = 46, 54 (*Souris*^*−/−*^ with, without hk *Mt*) cells examined over *n* = 3 independent experiments. *P* values were calculated using one-way ANOVA with Tukey’s post hoc. **i**, Ratio of intracellular itaconate measured by LC–MS in 24 h heat-killed *Mt* treated Souris^*−/−*^ versus Souris^*+/−*^ BMDMs. Bar graph shows a mean ± s.d. of *n* = 3 independent experiments. *P* values were calculated using unpaired, two-sided Student’s *t*-test. Source data

**Extended Data Fig. 2:**
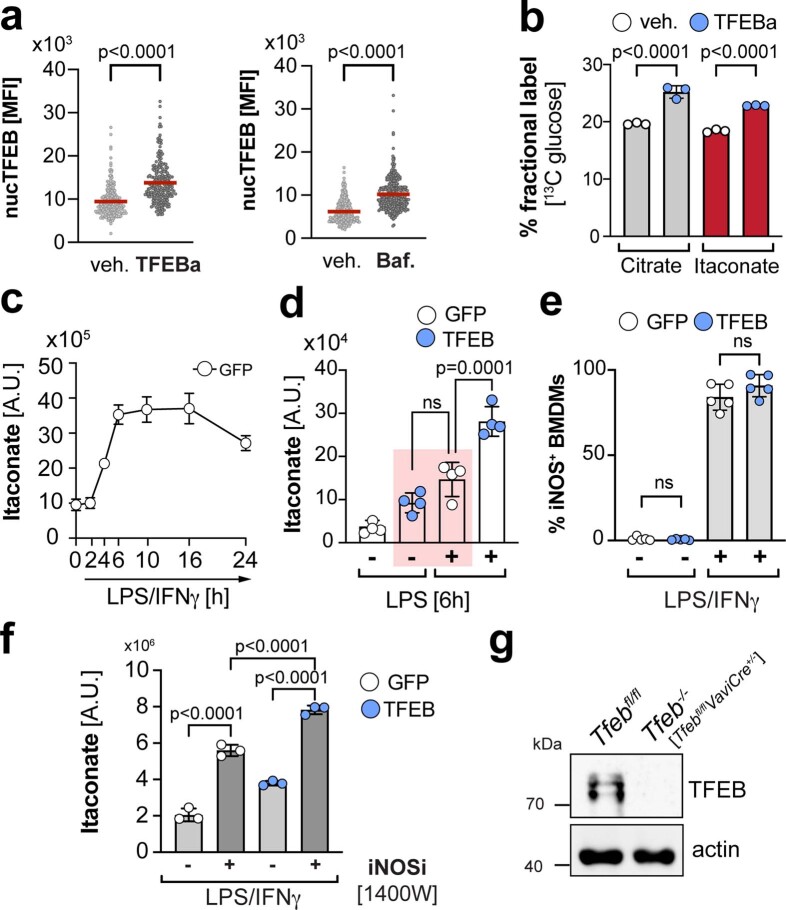
TFEB activation induces itaconate synthesis. **a**, Quantification of nuclear TFEB levels upon TFEB activation with TFEBa- or Baf-treatment in naïve BMDMs. Related to images in Fig. 2b,c. Graph shows nuclear TFEB levels of N = 240/207 (veh/TFEBa) and N = 326/315 (veh/Baf) individual cells examined over *n* = 2 independent experiments. *P* values were calculated using unpaired, two-sided Student’s *t*-test. **b**, TCA cycle and itaconate fueling determined by metabolic labelling of TFEBa- and vehicle-treated BMDMs with ^13^C-glucose. Results are based on GC-MS/MS measurements. Bars show mean ± s.d. of *n* = 3 biologically independent samples. *P* values were calculated using one-way ANOVA with Tukey’s post hoc. **c**, Intracellular itaconate levels measured by LC-MS in BMDMs treated with LPS/IFNy for the indicated time frames. Line graph shows mean ± s.e.m. of *n* = 5 biologically independent samples **d**, Intracellular itaconate levels in naïve and 6 h LPS-treated GFP- or TFEB-GFP-expressing BMDMs. Bar graph shows mean ± s.d. of *n* = 4 biologically independent samples. P-values were calculated using one-way ANOVA with Tukey’s post-hoc. n.s. with *P* > 0.05 **e**, iNOS expression determined by flow cytometry, in TFEB- and GFP-expressing BMDMs treated or not with LPS/IFNγ for 24 h. Bars represent mean ± s.d. of *n* = 3 independent experiments. *P* values were calculated using one-way ANOVA with Tukey’s post-hoc, n.s. with *P* > 0.05. **f**, Itaconate levels measured by LC/MS from 24 h LPS/IFNγ-treated GFP- or TFEB-expressing cells, treated or not with the iNOS inhibitor 1400 W. Bars represent mean ± s.d. of *n* = 3 biologically independent samples. *P* values were calculated using one-way ANOVA with Tukey’s post-hoc. **g**, Western Blot against endogenous TFEB in TFEB-deficient (*Tfeb*^*−/−*^) and respective control BMDMs. Source data

TFEB’s altered carbon funnelling resulted in substantially elevated cellular itaconate levels (Fig. 2a), as measured by liquid chromatography coupled to mass spectrometry (LC–MS). Similar to the genetic model, the activation of endogenous TFEB by the TFEB activator (TFEBa) 2-hydroxypropyl-β-cyclodextrin^24^ enhanced glucose-derived carbon fuelling of itaconate and increased itaconate levels (Fig. 2b and Extended Data Fig. 2a,b). Itaconate production was also induced by TFEB activation after lysosomal inhibition via the V-ATPase inhibitor bafilomycin A1 (Baf)^25^ (Fig. 2c and Extended Data Fig. 2a). Thus, TFEB activation alone is sufficient to produce itaconate without additional need for a pro-inflammatory macrophage signal.

Next, we examined the functional relationship between TFEB activation and itaconate production in macrophages challenged with bacterial stimuli. In control BMDMs, LPS/IFNγ stimulation caused peak levels of itaconate within the first 6 h of macrophage activation (Extended Data Fig. 2c). These peak levels were comparable to TFEB-induced itaconate levels in naïve macrophages (Fig. 2d, red rectangle). Additional activation of TFEB by LPS/IFNγ treatment or LPS signalling alone further augmented itaconate levels (Fig. 2d and Extended Data Fig. 2d), without affecting iNOS expression, a negative regulator of itaconate synthesis in activated BMDMs (Extended Data Fig. 2e,f)^26–28^. Conversely to TFEB-activated cells, BMDMs that were genetically depleted of TFEB (*Tfeb*^*−/−*^) (Extended Data Fig. 2g) were significantly impaired in itaconate synthesis in response to several heat-killed bacteria, including *Staphylococcus aureus*, *M. tuberculosis* and *S. enterica* serovar Typhimurium (Fig. 2e) and to LPS/IFNγ treatment (Fig. 2f). Thus, our data identify TFEB as a new driver of itaconate production during bacterial stimulation.

We also investigated the TFEB-itaconate pathway in macrophages from a murine model of the human immunodeficiency Chediak Higashi Syndrome (*Souris*). In this disorder, a key step for TFEB activation upon bacterial uptake, the maturation of the phago-lysosome, is disturbed^5,29^. Accordingly, we found that in diseased (*Souris*^*−/−*^) macrophage*s* nuclear TFEB translocation and itaconate synthesis were reduced relative to healthy macrophages (*Souris*^*+/−*^) treated with bacterial particles (Fig. 2g–i). Together, our data identify TFEB as novel regulator of itaconate production and provide evidence that phago-lysosomal dynamics and lysosomal stress signalling control the production of the mitochondrial metabolite itaconate.

**Fig. 3:**
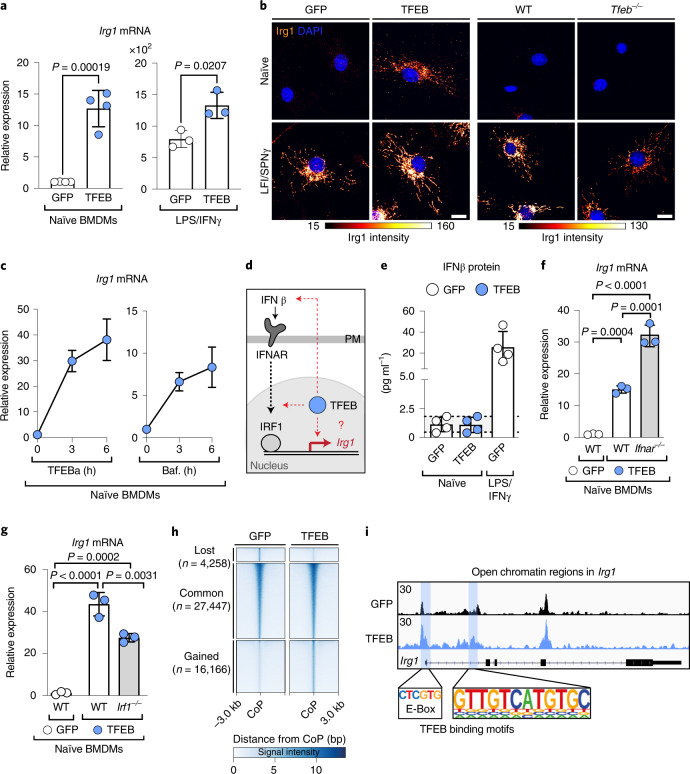
TFEB activation induces transcription of *Irg1*. **a**, Relative *Irg1* mRNA expression determined by real-time qPCR in naïve or 6 h LPS/IFNγ-treated TFEB-GFP- or GFP-expressing BMDMs. Bars show mean ± s.d. of *n* = 4 (left) *n* = 3 (right) independent experiments. *P* values were calculated using unpaired, two-sided Student’s *t*-test. **b**, Images of endogenous Irg1 visualized by immunofluorescence and treated without or with LPS/IFNγ for 6 h in (left) WT BMDMs expressing GFP- or TFEB-GFP, or (right) *Tfeb*^*−/−*^ and control BMDMs. Images are representative of *n* = 3 independent biological experiments. Scale bar, 10 µm. **c**, *Irg1* mRNA expression in BMDMs treated with 5 mM TFEBa or 100 nM Baf. Line graphs show the mean ± s.e.m. of *n* = 3 (TFEBa) and *n* = 5 (Baf) independent experiments. **d**, Schematic of potential mechanisms of TFEB-driven *Irg1* expression. **e**, Quantification of secreted IFNβ protein from naïve TFEB-GFP and GFP-expressing BMDMs. LPS/IFNγ-treated, GFP-expressing BMDMs served as positive control. Bars show mean ± s.d. of *n* = 4 independent experiments. **f**,**g**, Relative *Irg1* mRNA expression in naïve WT, *Ifnar1*^*−/−*^ (**f**) or *Irf1*^*−/−*^ (**g**) BMDMs, expressing TFEB-GFP or GFP. Bars show mean ± s.d. of *n* = 3 independent experiments. *P* values were calculated using one-way ANOVA, with Tukey’s post hoc. **h**, Heatmap depicting differentially accessible regions in GFP- and TFEB-GFP-expressing BMDMs, using a window of ±3 kb from the centre of the peak (CoP). Three clusters are represented denoting the commonly (common) accessible sites and the regions that loose or gain accessibility upon TFEB expression (lost and gained, respectively). **i**, Representative gene tracks from ATAC-seq data of the *Irg1* gene region. Blue boxes indicate significantly gained peaks in TFEB-GFP- relative to GFP-expressing BMDMs. The *y* axis represents the reads per kilobase of transcript per million of mapped reads. Potential TFEB binding sites, derived from motif analysis are highlighted. Data show *n* = 1 experiment with *n* = 2 technical repeats. Source data

**Extended Data Fig. 3:**
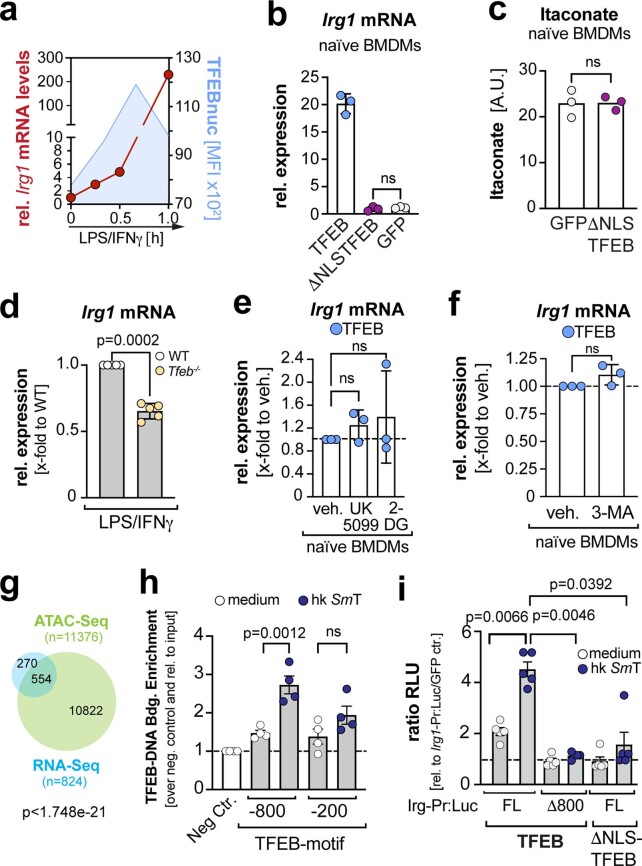
TFEB activation drives Irg1 expression independent of its metabolic targets and autophagy. **a**, Temporal correlation of *Irg1* mRNA expression from qRT-PCR (red) and nuclear TFEB levels from immunofluorescence imaging (blue) in BMDMs stimulated with LPS/IFNγ. Line curves show mean of *n* = 1 independent experiment. **b**, *Irg1* mRNA expression in naïve *Tfeb*^*−/−*^ BMDMs, expressing TFEB-GFP, GFP or ΔNLS-TFEB-GFP. Graph shows mean ± s.d. of *n* = 3 independent experiments. *P* values were calculated using one-way ANOVA with Tukey’s post-hoc, n.s. *P* > 0.05. **c**, Itaconate levels measured by LC-MS in GFP- or ΔNLS TFEB-GFP-expressing BMDMs. Bar shows mean of *n* = 3 independent experiments. *P* values were calculated using unpaired, two-sided Student’s *t*-test. n.s. for *P* > 0.05. **d**, *Irg1* mRNA expression in *Tfeb*^*−/−*^ and WT BMDMs treated for 6 h with LPS/IFNγ. Graphs show mean ± s.d. of *n* = 5 independent experiments. *P* values were calculated using two-tailed, one-sample *t*-test. **e,f**, *Irg1* mRNA expression in naïve TFEB-GFP-expressing BMDMs treated with vehicle, (**e**) 4 µM UK5099, 10 mM 2-DG, or (**f**) 10 mM 3-MA for 24 h. Graphs show mean ± s.d. of *n* = 3 independent experiments. *P* values were calculated using (**e**) one-way ANOVA with Tukey’s post-hoc and (**f**) two-tailed, one-sample *t*-test. n.s. for *P* > 0.05. **g**, Venn diagram depicting differentially expressed genes (p ≤ 0.01) from RNA-seq analysis (blue) from Fig. 1d, or genes annotated to differentially accessible chromatin regions from ATAC-seq (green) *n* = 1 independent experiment with N = 2 replicates and the intersections of these data sets in naïve TFEB-GFP- versus GFP-expressing BMDMs. Significance of overlap was calculated with hypergeometric statistical test. **h**, ChIP-qPCR of iBMDMs stably expressing human TFEB-GFP treated with or without heat-killed *Sm*T (10^5^ particles / mL) for 90 min. Bars show mean ± s.e.m. from *n* = 4 independent biological experiments. *P* values were calculated using one-way ANOVA with Tukey’s post-hoc. n.s. for *P* > 0.05. **i**, Mouse embryonic fibroblast expressing the indicated TFEB and *Irg1*-promoter-Luciferase constructs were incubated with medium or heat-killed *Sm*T (10^5^ particles / mL) for 3 h and fold induction of firefly luciferase levels (RLU) was determined in TFEB/*Irg1*-promoter vs. GFP/*Irg1*-promoter expressing cells. Bar shows mean ± s.e.m. of *n* = 5 independent experiments. *P*values were calculated using one-way Welch’s ANOVA with Dunnett’s post hoc. n.s. for *P* > 0.05. Source data

Itaconate synthesis depends on the enzymatic activity of Irg1 (ref. ^30^). Given the comparable timeline between nuclear TFEB translocation and the increase of *Irg1* messenger RNA levels (Extended Data Fig. 3a), we hypothesized that TFEB controls *Irg1* expression. Indeed, stimulation through wild-type (WT) TFEB expression, but not its ΔNLS-mutant (Extended Data Fig. 3b,c), induced *Irg1* on the mRNA and protein level (Fig. 3a,b left panel). Similarly, pharmacological activation of endogenous TFEB induced *Irg1* mRNA levels (Fig. 3c). In contrast, *Irg1* levels were significantly reduced in *Tfeb*^*−/−*^ macrophages (Fig. 3b, right panel and Extended Data Fig. 3d). Thus, TFEB activity controls *Irg1* expression.

Two mechanisms were recently shown to promote *Irg1* expression: type I interferon signalling and interferon regulatory factor 1 (IRF1) activation^31,32^. However, their roles for TFEB-driven *Irg1* expression are unclear (Fig. 3d). We demonstrate that TFEB activation neither induced the expression of interferon β, nor did the loss of the interferon receptor (IFNAR1) suppress TFEB-driven *Irg1* transcription (Fig. 3e,f). Similarly, TFEB was able to elevate *Irg1* expression in BMDMs lacking IRF1, albeit total *Irg1* mRNA levels were reduced (Fig. 3g). Thus, our data show that TFEB contains an inherent ability to increase *Irg1* mRNA levels, which is additionally modulated by previously described type I interferon elements^26,27^. Beyond that, glycolysis^33^, mitochondrial pyruvate-import and the TFEB-target autophagy^34^ (Extended Data Fig. 3e,f) were dispensable for *Irg1* expression.

To address the possibility that TFEB directly engages *Irg1* expression, we examined chromatin accessibility changes by assay for transposase-accessible chromatin with high-throughput sequencing (ATAC-seq). Comparing our ATAC- and RNA-seq data showed that changes in chromatin accessibility correlated significantly with changes in gene expression (Extended Data Fig. 3g). Overall, we found 16,000 more accessible chromatin sites in TFEB-activated macrophages, including two sites in the promoter region of the *Irg1* gene (Fig. 3h,i blue squares). Motif analysis in open *Irg1* regions revealed, amongst others (Supplementary Table 1), TFEB-consensus sites (Fig. 3i, lower panel), suggesting that TFEB may directly target *Irg1* transcription. Supporting this, chromatin immunoprecipitation–quantitative PCR (ChIP–qPCR) showed that TFEB binds to the *Irg1* promoter at an element 800 bp upstream of the transcriptional start site (Extended Data Fig. 3h). This region, containing the TFEB-consensus motif, was also essential for nuclear TFEB-driven expression from the *Irg1* promoter, as assayed by a luciferase-*Irg1*-promoter system (Extended Data Fig. 3i). Thus, our data support a model whereby TFEB directly induces *Irg1* expression to enhance the production of the antimicrobial metabolite itaconate.

**Fig. 4:**
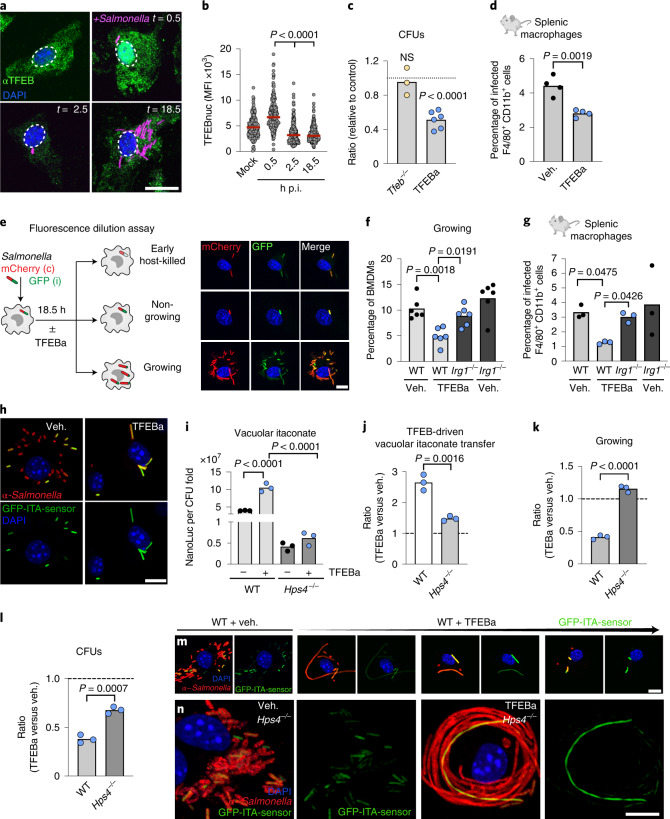
TFEB-driven itaconate production reduces intracellular *Salmonella* growth in infected macrophages. **a**, Indirect immunofluorescence against endogenous TFEB. Scale bar, 15 µm. **b**, Quantification of nuclear TFEB levels from **a**. Graph shows nuclear TFEB mean fluorescence intensity from *n* = 170 cells examined over *n* = 3 independent experiments. *P* values were calculated using one-way ANOVA with Dunnett’s post hoc. **c**, Ratio of CFUs of *Tfeb*^*−/−*^ to WT or TFEBa relative to vehicle-treated BMDMs from *n* = 3 (*Tfeb*^*−/−*^) or *n* = 6 (TFEBa) independent experiments. *P* values were calculated using two-tailed one-sample *t*-test, NS *P* > 0.05. **d**, Percentage of infected splenic macrophages treated with TFEBa or PBS. Bars show mean from *n* = 4 mice and *P* values were calculated using unpaired, two-sided Student’s *t*-test. **e**, Strategy to identify different *Salmonella* subpopulations inside macrophages and corresponding images. Scale bar, 10 µm. **f**, Percentage of cells with growing *Salmonella* in WT or *Irg1*^*−/−*^ BMDMs treated or not with TFEBa and analysed by flow cytometry. Bars show mean from *n* = 6 independent experiments. *P* values were calculated using one-way ANOVA with Tukey’s post hoc. **h**, Percentage of infected splenic macrophages of TFEBa- or PBS-treated WT or *Irg1*^*−/−*^ mice. Bars show mean from *n* = 3 mice. *P* values were calculated using one-way Welch’s ANOVA, with Dunnett’s post hoc test. **h**, Images of BMDMs infected with GFP-itaconate sensor-carrying *Salmonella*, treated or not with TFEBa for 18.5 h. Scale bar, 10 µm, images are representative of *n* = 4 independent experiments. **i**,**j**, Luciferase measurements from NanoLuc-ITA-*Salmonella*-infected BMDMs (normalized to fold-change CFU) (**i**) and as ratio of TFEBa- to vehicle-treated BMDMs (**j**). Graphs show mean of *n* = 3 independent biological experiments. *P* values were calculated using one-way ANOVA with Tukey’s post hoc test (**i**) and unpaired, two-sided Student’s *t*-test (**j**). **k**, Ratio of cells containing growing bacteria in TFEBa and vehicle-treated WT or *Hps4*^*−/−*^ BMDMs (based on Extended Data Fig. 5g). Bars show mean of *n* = 3 independent experiments. *P* values calculated using unpaired, two-sided Student’s *t*-test. **l**, Ratio of CFUs in TFEBa and vehicle-treated WT or *Hps4*^*−/−*^ BMDMs. Dashed line indicates vehicle-treated control level. Bars show mean of *n* = 3 independent experiments. *P* values were calculated using unpaired, two-sided Student’s *t*-test. **m**,**n**, Images of GFP-ITA-*Salmonella*-infected WT and *Hps4*^*−/−*^ BMDMs, treated or not with TFEBa. Images are representative of *n* = 3 independent experiments. Scale bars, 10 µm. Source data

**Extended Data Fig. 4:**
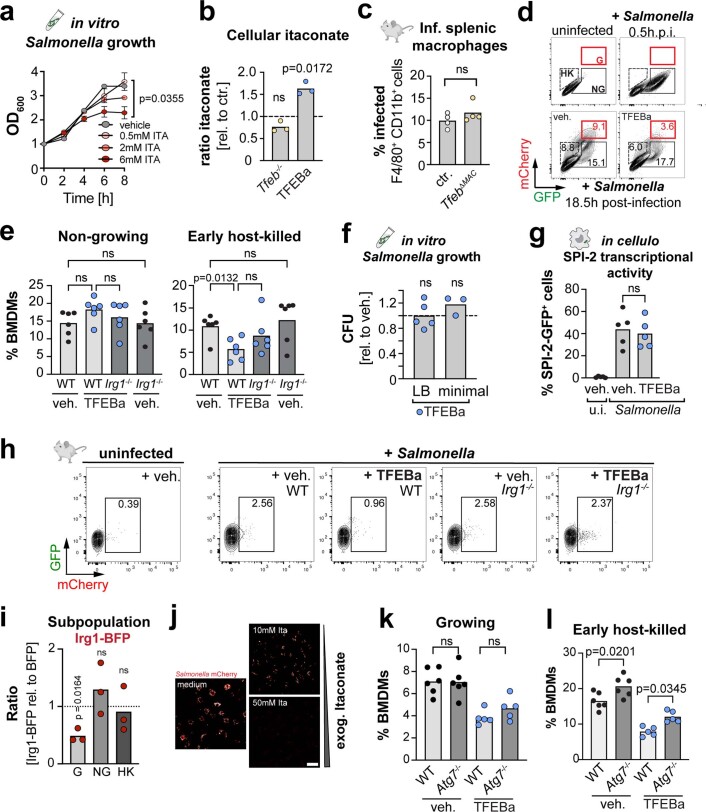
TFEB drives itaconate synthesis in infected macrophages to restrain intracellular *Salmonella* proliferation. **a**, *Salmonella* growth measured in OD_600_, in the presence of increasing itaconate concentration. Data show mean ± s.e.m. from *n* = 3 independent biological experiments. *P* values of the 8 h time points were calculated using one-way ANOVA with Dunnett’s post-hoc. **b**, Ratios of cellular itaconate of *Tfeb*^*−/−*^ to WT or TFEBa- to vehicle-treated BMDMs. Bars show mean of *n* = 3 independent biological experiments. *P* values were calculated using two-tailed one-sample *t*-test, n.s. *P* > 0.05. **c**, Percentage of infected macrophages in spleens of *Tfeb*^*ΔMac*^ or control mice, three days after i.p. *Salmonella* infection. Bar shows mean of *n* = 4 mice. *P* values were calculated using unpaired, two-sided Student’s *t*-test. n.s. with *P* > 0.05**. d,e**, Flow cytometry analysis of *Salmonella* subpopulations upon TFEBa- or vehicle- treatment for 18.5 h in WT or *Irg1*^*−/−*^ BMDMs. (e) Quantification of (d). Graphs show mean from *n* = 6 independent experiments. *P* values were calculated using one-way ANOVA with Tukey’s post-hoc. n.s. for *P* > 0.05. **f**, CFUs of *Salmonella* grown in vitro in LB or minimal medium for 18.5 h with or without TFEBa. CFUs are shown as ratio of TFEBa- to vehicle-treated control samples. Bar graphs show mean ± s.d. of *n* = 5 (LB) and *n* = 3 (minimal) independent experiments. *P* values were calculated using two-tailed one-sample *t*-test. n.s. for *P* > 0.05. Dashed lines indicate respective vehicle-treated control cells. **g**, SPI-2-activation assayed by flow cytometry. BMDMs infected with *Salmonella* carrying a SPI-2-GFP-reporter and treated with or without TFEBa. Bar graphs show mean percentage of GFP-positive BMDMs from *n* = 5 independent biological repeats. *P* values were calculated using one-way ANOVA with Tukey’s post-hoc, n.s. *P* > 0.05. **h**, Flow cytometry of *Salmonella*-mCherry-containing splenic macrophages of WT or *Irg1*^*−/−*^ mice i.p. infected and treated with TFEBa or PBS. Plots are representative of *n* = 3 mice. **i**, Ratios of *Salmonella* subpopulations in Irg1-BFP- relative to BFP-expressing BMDMs. Bars show mean from *n* = 3 independent experiments. *P* values were calculated using two-tailed one-sample *t*-test. n.s. for p > 0.05. **j**, Bacterial load in *Salmonella*-infected BMDMs treated with exogenous itaconate for 18.5 h. Images are representative of *n* = 3 independent experiments. Scale bar: 50 µm. **k,l**, Percentage of BMDMs containing (**k**) growing or (**l**) early host-killed *Salmonella* in WT and autophagy-deficient *Atg7*^*−/−*^ BMDMs, treated with TFEBa or vehicle. Data represent mean of *n* = 6 (veh) and *n* = 5 (TFEBa) independent experiments. *P* values were calculated using one-way ANOVA with Tukey’s post-hoc, n.s. for *P* > 0.05. Source data

TFEB activation and itaconate synthesis have both individually been suggested to restrict the survival of several intracellular bacteria^18,35^ (Extended Data Fig. 4a). However, these two cellular processes have only been viewed as separate pathways and were never functionally linked as part of a concerted antibacterial response. To address the functional role of the TFEB–Irg1–itaconate pathway for the survival of an intracellular bacterium, we infected macrophages with the food-borne facultative intracellular pathogen, *S. enterica* serovar Typhimurium. This itaconate-sensitive pathogen^23^ was shown to escape TFEB control by inactivating the transcription factor shortly after infection^36^, which we confirmed by measurements of reduced cellular and nuclear TFEB levels (Fig. 4a,b). Supporting that *Salmonella* efficiently restrict TFEB activity, itaconate levels were only mildly reduced in infected *Tfeb*^*−/−*^ BMDMs (Extended Data Fig. 4b) and bacterial loads were comparable between WT and *Tfeb*-deficient macrophages in vitro and in vivo (Fig. 4c and Extended Data Fig. 4c). In contrast, the TFEBa was able to overcome bacteria-induced TFEB repression: it raised cellular itaconate levels (Extended Data Fig. 4b) and lowered bacterial loads in macrophages in vitro and in vivo (Fig. 4c,d), corroborating beneficial effects of this treatment in *Salmonella*-infected mice^37^.

To understand in detail how the TFEB-itaconate pathway targets intracellular *Salmonella* survival, we made use of an established *Salmonella* fluorescence dilution system. This experimental system allows the distinction between different macrophage control mechanisms: the ability of the macrophage to (1) kill the pathogen and (2) restrict its escape into proliferating states^38^. For this, we infected macrophages with *Salmonella* that carried the fluorescence dilution-plasmid with constitutively expressed mCherry and conditionally expressed GFP, whose synthesis is terminated at the onset of infection (Fig. 4e). Proliferating bacteria display high mCherry to GFP signal intensity (Fig. 4e and Extended Data Fig. 4d growing, red gate) and can be distinguished from non-proliferating bacteria (constant mCherry and GFP signals) (Fig. 4e and Extended Data Fig. 4d, non-growing, black gate), early host-killed bacteria (mCherry-positive, but low GFP signal) (Fig. 4e and Extended Data Fig. 4d host-killed, dashed gate) and later degradation stages that lose all fluorescence.

Using this fluorescence dilution assay, we found that pharmacological TFEB activation led to a specific and marked reduction of the proliferating *Salmonella* population (Fig. 4f and Extended Data Fig. 4d), with milder or non-significant effects on the other two bacteria subsets (Extended Data Fig. 4d,e). A direct effect of TFEBa on *Salmonella* proliferation and survival could be ruled out, as this treatment did not reduce the in vitro growth of bacteria when cultured in complete or a minimal bacterial growth medium that mimics the vacuolar environment of macrophages^39^ (Extended Data Fig. 4f). Also, the activation of the *Salmonella* pathogenicity island 2 (SPI-2) virulence programme, which is essential for intracellular bacterial proliferation^40^, was grossly unaffected by TFEBa treatment (Extended Data Fig. 4g). Instead, we found that the antiproliferative and bacterial load-reducing effect in macrophages upon TFEB re-activation was dependent on Irg1-driven itaconate synthesis, as shown by experiments in *Irg1*^*−/−*^ BMDMs and mice (Fig. 4f,g and Extended Data Fig. 4h). Moreover, ectopic *Irg1* expression (Extended Data Fig. 4i) and extracellular addition of itaconate to infected macrophages (Extended Data Fig. 4j) both reduced the growing bacterial subset, corroborating the repressive effect of itaconate on *Salmonella* proliferation. Bacterial repression by the TFEB-itaconate axis was largely independent of autophagy, another TFEB-induced pathway^34,41^, as assessed in autophagy-deficient *Atg7*^*−/−*^ BMDMs (Extended Data Fig. 4k). Instead, autophagy contributed significantly to the early host-killed population in TFEBa-treated and control macrophages (Extended Data Fig. 4l), as suggested previously^37^. Thus, our data identify itaconate production as a new Atg7-independent TFEB-executor, selectively controlling the virulent subset of proliferating *Salmonella*.

**Extended Data Fig. 5:**
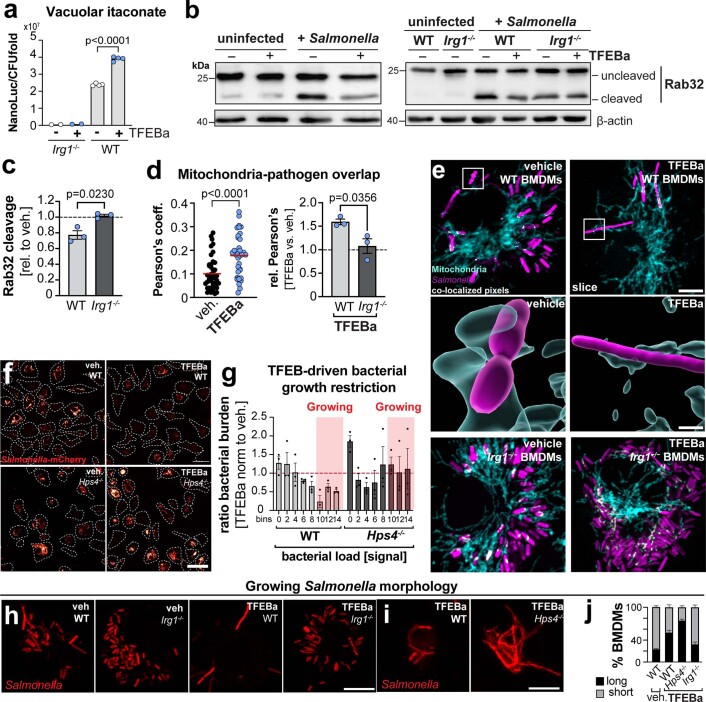
TFEB increases vacuolar itaconate levels by modulating Rab32/BLOC3 transfer system. **a**, Vacuolar itaconate levels in WT or *Irg1*^*−/−*^ BMDMs infected with *Salmonella* carrying a NanoLuc-itaconate sensor and treated with TFEBa or vehicle for 18 h. Graphs show mean of *n* = 4 (WT) independent experiments or n = 2 biologically independent samples (*Irg1*^*−/−*^). *P* values were calculated using one-way ANOVA with Tukey’s post-hoc **b,c**, Levels of Rab32 cleavage in WT or *Irg1*^*−/−*^ BMDMs infected with *Salmonella* (*Sm*T) and treated or not with TFEBa. (**b**) Representative Western Blots and (**c**) quantification of Rab32 cleavage in infected TFEBa- relative to vehicle-treated WT and *Irg1*^*−/−*^ BMDMs. Bars show mean ± s.d. from *n* = 3 independent experiments. *P* values were calculated using unpaired, two-sided Students t-test. **d,e**, Co-localization of mitochondria with growing *Salmonella*. Data show (**d**) calculated Pearson’s coefficients and coefficient ratio of TFEBa- to vehicle-treated controls in WT and *Irg1*^*−/−*^ BMDMs. Left graph: data present N = 44 (veh,) N = 35 (TFEBa) individual cells examined over *n* = 3 independent experiments. red line: mean. Right graph: Bars shows mean ± s.d. of *n* = 3 independent experiments. *P* values were calculated using two-sided, unpaired Student’s t-test. (**e)** Representative images from (**d**). Inserts show 3D-rendered surfaces of mitochondria and *Salmonella*. Scale bars: 5 µm and insert 1 µm. **f,g**, Images of bacterial load in mCherry-*Salmonella*–infected WT and *Hps4*^*−/−*^ BMDMs, treated or not with TFEBa. (**f**) Representative images of *n* = 3 independent experiments. Scale bar: 40 µm, dashed lines indicate cellular outlines. (**g**) Frequency distribution of (**f**) shown as ratio of TFEBa- to vehicle-treated BMDMs. Bars represent mean ± s.e.m. from *n* = 3 independent experiments. Red box shows bins containing BMDMs with growing *Salmonella* populations. **h,i**, Images of BMDMs containing *Salmonella* with filamented morphology. Specified BMDMs were treated with the TFEBa or vehicle for 18.5 h. **j**, Analysis of (h,i). Percentage of BMDMs with filamented (long) and short Salmonella. Data show mean ± s.e.m. of *n* = 5 (*Irg1*^*−/−*^*)* and *n* = 3 (*Hps4*^*−/−*^) independent experiments. Source data

Itaconate can be transferred from mitochondria into the pathogen-containing vacuole via Irg1-Rab32–BLOC3-driven organelle interactions and directly inhibit *Salmonella* growth^9,23^ (Extended Data Fig. 4a). To assess whether vacuolar itaconate mediates the growth suppressive TFEB-effect, we infected macrophages with *Salmonella* carrying previously described sensor plasmids (GFP-ITA or NanoLuc-ITA) that allow the visualization and measurement of vacuolar itaconate^9^ (Fig. 4h and Extended Data Fig. 5a). Although, *Salmonella* Typhimurium can partially counteract vacuolar itaconate transport by cleaving Rab32 (ref. ^9^) (Extended Data Fig. 5b), we found that TFEB activation increased the percentage of itaconate-exposed pathogens (Fig. 4h) and raised vacuolar itaconate levels by 2.6-fold in comparison to control cells (Fig. 4i,j). TFEB activation was accompanied by a mild, *Irg1*-dependent reduction in Rab32 cleavage and slightly increased mitochondria-pathogen colocalization (Extended Data Figs. 5b–e), supporting that Irg1 is part of the Rab32–BLOC3 itaconate-transfer system, as reported previously^9^. Genetic inactivation of the Rab32–BLOC3-transfer system (*Hps4*^*−/−*^ BMDMs*)*^9^ repressed TFEB’s ability to elevate vacuolar itaconate and decreased itaconate to similar levels found in *Hps4*^*−/−*^ control cells (Fig. 4i,j). This supports that TFEB depends on active Rab32–BLOC3 sites to increase vacuolar itaconate levels.

To assess in depth the influence of vacuolar itaconate transfer on bacterial proliferation in macrophage populations, we quantified the bacterial load per cell by single cell imaging (Extended Data Fig. 5f,g). TFEB-itaconate activation in WT BMDMs specifically targeted macrophages with high bacterial burden and lowered the number of proliferating bacteria in these cells (Fig. 4k and Extended Data Fig. 5f,g). In striking contrast, TFEB activation in *Hps4*^*−/−*^ cells failed to restrain bacterial proliferation (Fig. 4k and Extended Data Fig. 5f,g), which was also mirrored by an increase in bacterial colony-forming units (CFUs) (Fig. 4l). Thus, our data indicate that TFEB elevates itaconate synthesis upon which the metabolite is transferred into the vacuole in a Rab32 or Hps4-dependent manner to inhibit pathogen proliferation.

Modulating vacuolar itaconate levels downstream of TFEB activation, our imaging analysis revealed gradual effects on bacterial growth and division: (1) unperturbed bacterial proliferation (growth and division) occurred in the absence of itaconate (*Irg1*^*−/−*^) (Fig. 4f, Extended Fig. 5h), (2) restriction of bacterial proliferation when TFEB-driven itaconate was successfully transferred into the vacuole (WT) (Fig. 4h,m) and (3) pathogens formed filaments, a state in which the microbe grew, but ceased to divide, when vacuolar itaconate was substantially lowered (*Hps4*^*−/−*^, and TFEB escapees) (Fig. 4m,n and Extended Data Fig. 5i,j). This bacterial filamentation may contribute to the partially lowered numbers of CFUs in TFEBa-treated *Hps4*^*−/−*^ cells (Fig. 4l). Thus, our data support a role for TFEB-driven vacuolar itaconate in blocking intra-macrophage proliferation of *Salmonella*.

Our findings expand the function of TFEB beyond its established regulatory roles for lysosome biology and autophagy^34^. While TFEB has been linked to the control of metabolic functions in non-immune cells^41,42^, the TFEB–Irg1–itaconate pathway has remained undetected, probably due to the restricted expression of Irg1 by only few cell types^43,44^. In primary macrophages, our results identify the TFEB-driven Irg1–itaconate axis as a lysosome-to-mitochondria communication pathway that controls a cell-autonomous antibacterial defence mechanism to protect the phago-lysosomal compartment from being exploited as bacterial proliferation niche. Our data indicate that the TFEB–Irg1–itaconate pathway exerts its antimicrobial function primarily in the vacuole: functions of cytosolic itaconate^45,46^, TFEB or Hps4, and potential functional interactions between those systems^47^, may play additional roles or modulate the vacuolar response^48^. How itaconate controls *Salmonella* proliferation mechanistically is currently unclear, but probably relates to its ability to inhibit selected metabolic enzymes. This includes key enzymes in the glyoxylate shunt and propionate metabolism that different pathogens rely on for intra-macrophage growth during acute and chronic infections^23,35,49–51^. Given the reported reduced itaconate synthesis in human macrophages^4^, it remains to be determined whether the TFEB–Irg1–itaconate pathway also holds promise to target virulent infections with *Salmonella* Typhimurium, or other itaconate-sensitive microbes such as *S*. Typhi and *Mycobacterium* spp.^9,35^ in humans. Beyond its antimicrobial activity itaconate is also known as an immunomodulatory metabolite^45,52^. Thus, one could speculate that some of TFEB’s reported anti-inflammatory and immunomodulatory effects may act through control of itaconate^18,53^.

## Methods

### Antibodies and reagents

The following primary antibodies and dyes were used for immunofluorescence staining (IF) or Western Blot (WB): anti-IRG1 (IF 1:100; Abcam, ab222411), anti-HSP60 (IF 1:1,000; CST, MA5-15836), anti-TFEB (WB: 1:3,000; IF: 1:1,000; Bethyl Laboratories, A303-673A), anti-*Salmonella* Typhimurium control serum (IF 1:10,000; TS1624, Sifin), anti-Rab32 (WB 1:2,000, LS-C204248, LSBio), anti-actin (WB 1:10,000; SantaCruz, sc-47778). The following secondary antibodies were used: antirabbit HRP-linked (WB 1:10,000; CST, 7074), antigoat HRP-linked (WB 1:10,000; ThermoFisher, 31402), antirabbit Alexa Fluor 647-conjugated (IF 1:500; ThermoFisher, A-21244) and antirabbit Cy3-conjugated (IF 1:1,000; Jackson Immuno Research Laboratories, 111-165-144). LysoTracker Red DND-99 (L7528) was purchased from ThermoFisher. The following stimuli were used: IFNγ (50 ng ml^−1^; PeproTech, AF-315-05), LPS (20 or 100 ng ml^−1^; InvivoGen, tlrl-pb5lps), macrophage colony stimulating factor (20 ng ml^−1^; PeproTech, 315-02), heat-killed *M. tuberculosis* (hk *Mt*; 10 µg ml^−1^; InvivoGen, tlrl-hkmt), heat-killed *S. aureus* (HKSA; 10^6^ particles per ml; InvivoGen, tlrl-hksa), heat-killed *Salmonella* Typhimurium (HKST; five particles per BMDM; InvivoGen, tlrl-hkst2). The following chemicals were purchased from Sigma Aldrich: TFEBa (2-hydroxypropyl-β-cyclodextrin 5 mM; H-107); 2-deoxy-d-glucose (2-DG; 10 mM, D6134), 3-methyladenine (3-MA; 10 mM; M9281), l-arabinose (10839), Bafilomycin A1 (Baf, 100 nM, B1793), mouse serum (M5905) and UK5099 (4 µM, PZ0160).

### Mice

Mice were maintained in specific pathogen-free conditions under protocols approved by the animal care committee of the Regierungspräsidium Freiburg, Germany, in compliance with all relevant ethical regulations. Mice were housed under controlled conditions, namely 20–21 °C, 55–65% relative humidity and 12/12 h light/dark cycle. Food was available ad libitum for all animals. Eight- to 22-week-old animals were euthanized by carbon dioxide asphyxiation followed by cervical dislocation, and bone marrow or spleens were harvested postmortem.

The following mice were used: C57BL/6J, *Tfeb*^*fl/fl*^ *Vav-iCre* or *Tfeb*^*fl/fl*^ *or Lyz2-Cre* mice. *Tfeb*^*fl/fl*^ mice^17^ were kindly provided by A. Ballabio (Faculty of Medicine, Frederico II University of Naples, Italy). *Irg1*^*−/−*^ mice (C57BL/6NJ-Acod1^em1(IMPC)J^/J), *Irf1*^*−/−*^ mice (B6.129S2-*Irf1*^*tm1Mak*^/J) and *Hps4*^*−/−*^ mice (B6.C3-*Pde6b*^*rd1*^ *Hps4*^*le*^/J) were purchased from the Jackson Laboratories. *Ifnar1*^*−/−*^ mice (B6.129s2-Ifnar^tm(Neo)Agt^) and *Souris*^*−/−*^ mice (C57BL/6J-*Lyst*^*bg-Btlr*^/Mmucd) were kindly provided by P. Stäheli (Institute of Virology, University Clinics Freiburg, Germany) and P. Aichele (Institute for Microbiology and Hygiene, University Clinics Freiburg, Germany), respectively. K. Simons provided bones from *Atg7*^*fl/fl*^ Vav-iCre mice (Kennedy Institute of Rheumatology, University of Oxford). For most mice, sex- and age-matched control littermates were used. Sex-matched Cre-negative *Tfeb*^*fl/fl*^ littermates were used as control for experiments with TFEB-deficient cells, since tested biological responses were unaffected by the presence or absence of Cre.

In vivo infection studies of 12–25-week-old female and male mice (Fig. 4d,g and Extended Data Fig. 4c,d) were infected intra-peritoneally with 5 × 10^4^ CFU per mouse of *S.* Typhimurium SL1344 with arabinose-induced pFCcGi-Cb. Animals were kept for 3 days and TFEBa (0017; Acacetin, Sigma Aldrich) dissolved in PBS (20 mg kg^−1^, sonicated for 5 min) or relevant solvent (PBS) was injected daily. For Tfeb-deletion studies, mice with macrophage-specific Tfeb knockout were used (*Tfeb*^*fl/fl*^ *Lyz2-Cre*), annotated as *Tfeb*^Δmac^. Mice were euthanized by CO_2_ and cervical dislocation and spleens were harvested. Cell suspensions were obtained by homogenizing the spleens using 70-μm cell strainers. Erythrocyte lysis (ACK lysing buffer, Gibco A1049201) was performed and unspecific binding was blocked with anti-CD16/32 for 15 min before cells were stained for F4/80 (1:200, BM8, BioLegend, 123137), Cd11b (1:500, M1/70, eBioscience, 50-0112-82) and live/dead (1:200, eBioscience, 65-0866-14) in cold PBS (Gibco) for 1 h. Cells were fixed for 15 min using the Foxp3 transcription factor staining buffer set (eBioscience, 00-5523-00). Data was acquired on a LSR Fortessa (BD) and analysed with FlowJo software (BD, v.10). During analysis, doublets were excluded. For the gating strategy, please see Supplementary Fig. 1.

### BMDM culture

Bone marrow was isolated from femur, tibia and pelvic bone of 8–12-week-old male and female mice. BMDMs were differentiated in BMDM medium (RPMI 1640, 10% FCS, 100 U ml^−1^ penicillin and 0.1 mg ml^−1^ streptomycin) containing 20 ng ml^−1^ macrophage colony stimulating factor at 37 °C and 5% CO_2_. Cells were grown for 6 days and then gathered with 0.25% trypsin. For most experiments, BMDMs were plated in BMDM medium. For *Salmonella* infection assays, BMDMs were plated in BMDM infection medium (DMEM, 10% FCS, 10% L929 supernatant, 1 mM sodium pyruvate, 4 mM glutamine).

### Retrovirus

pBMN-TFEB-GFP and ΔNLS-TFEB-GFP plasmids were kind gifts from R. Youle and S. Ferguson, respectively^54,55^. The pBMN-Irg1-BFP plasmid was generated by replacing the *Tfeb* gene in pBMN-TFEB-GFP plasmid with the *Irg1* sequence from the pCMV6-Entry-Irg1-Myc-DDK-tagged plasmid purchased from Origene and the GFP was replaced by the blue fluorescent protein (BFP) sequence. New plasmids were generated using the CloneAmp HiFi PCR Premix and In-Fusion HD Cloning Kit (Takara) according to the manufacturer’s instructions. For production of viral particles, 2.5 × 10^6^ PlatE cells were plated and the following day transfected with pBMN-plasmid DNA using Lipofectamine 3000 according to the manufacturer’s instructions. Viral supernatant was collected every 24 h for 4 days.

### BMDM transduction and sorting

For BMDM transduction, viral particles (diluted 1:3 in BMDM medium) were added to the bone marrow culture on day 2 of BMDM differentiation. After 18 h, transduction medium was removed and cells were cultured for three additional days. Where necessary, virus-targeted BMDMs were sorted using the BD FACSAria III cell sorter FACSDiva (BD, v.8.0.1).

### RNA-seq

RNA isolation was performed using the RNeasy MinElute Cleanup Kit according to the manufacturer’s instructions. Complementary DNA libraries were prepared by the Deep Sequencing facility at the Max Planck Institute of Immunobiology and Epigenetics using the TrueSeq stranded mRNA protocol (Illumina) and sequenced on a HiSeq 3000 (Illumina) platform to a depth of 16 million reads per sample. Sequencing data were analysed using the Galaxy platform provided by the Bioinformatics Core Facility of the Max Planck Institute of Immunobiology and Epigenetics and the University of Freiburg. The STAR aligner^56^ was used for trimming and mapping, GRCm38 as the reference genome. Quantification of the mapped reads was performed with featureCounts^57^ (10.1093/bioinformatics/btt656) and differential gene expression determined using the DESeq2 algorithm^58^ (10.1186/s13059-014-0550-8). Expression data were further processed and filtered using R (Lucent Technologies). For biological pathway enrichment analysis, significantly upregulated genes (adjusted *P* ≤ 0.01) in TFEB-GFP- versus GFP-expressing BMDMs were subjected to the PANTHER classification system v.13 (http://www.pantherdb.org) using the Gene List analysis (Statistical overrepresentation test, Annotation Data Set, PANTHER GO-Slim Biological Process; Reference List, Default *Mus musculus* genes) to define overrepresented biological processes.

### ATAC-seq

BMDMs were collected with 0.25% trypsin and 50,000 BMDMs per sample were lysed in ice-cold lysis buffer (10 mM Tris-Cl, 10 mM NaCl, 3 mM MgCl_2_, 0.1% (v/v) Igepal CA-630, pH 7.4), immediately followed by centrifugation at 500*g*, 4 °C. Pellets containing BMDM nuclei were subjected to transposition reaction using the Nextera DNA Flex Library Prep Kit (Illumina). DNA libraries were sequenced in paired-end mode (75 cycles) on a HiSeq 3000 (Illumina) by the Deep Sequencing facility at the Max Planck Institute of Immunobiology and Epigenetics with a reading depth of 50 million reads per sample in two biological replicates per condition. ATAC-seq was run in two replicates per condition. Adapter sequences were trimmed with Trimmomatic (v.0.36)^59^ and the Bowtie2 (ref. ^60^) algorithm (v.2.1.0) using the «–very-sensitive» parameter for aligning ATAC-seq reads to the mouse genome version GRCm38/mm10. Samtools^61^ (v.0.1.19) were used for data filtering and file format conversion. Duplicate reads and chr M were removed before peak calling. MACS2 (ref. ^62^) (v.2.1.0) algorithm was used for ATAC-seq peak identification with a *P* value cut-off of 1 × 10^3^. Genomic regions that are common or different from a set of peak files were identified with BEDTools^63^ (v.v.2.25.0). All .bam files were converted to bedgraphs with genomeCoverageBed a subcommand of BEDTools^63^. Gene annotation (100 kb upstream and 50 kb downstream from the transcription start site) and genomic distribution of accessible regions identified by MACS2 (ref. ^62^) were performed with BEDTools^63^ and -closetBed and -intersectBed subcommands, respectively. Clustering of regions was generated with ComputeMatrix function of DeepTools^64^, using reference-point–referencePoint centre -b 3000 -a 3000 -R<bed files>-S<bigwig files> as parameters. The function plotHeatmap from the same package was used for displaying the average profiles heatmap. Differentially accessible chromatin regions were scanned for enriched short-sequence motifs using HOMER^65^ software with the ‘findMotifsGenome.pl’ command. To search for a set of sequences for occurrences of specific known motifs FIMO^66^ from the MEME suite^67^ was used. For the *Irg1* gene a window of 1 kb upstream and downstream from the start and end of the two significant gained narrow peaks was analysed with BEDTools 5 subcommand -slopBed -b 1000 and motif occurrences with a *P* value of less than 0.0001 were chosen.

### ChIP

ChIP was performed as previously described^68^. Briefly, for each ChIP experiment 8–10 million cells were cross-linked with 1% formaldehyde (Pierce) for 10 min at room temperature, nuclei were isolated and chromatin was sonicated at 4 °C using a Bioruptor (Diagenode) for 25 cycles (30 s ‘ON’ and 30 s ‘OFF’, power setting high). For each immunoprecipitation 18 μl of antibody against TFEB (Cell Signaling, no. 37785, D2O7D) were incubated with chromatin at 4 °C with rotation overnight. Chromatin was washed, crosslink was reversed at 65 °C overnight and DNA was isolated using Agencourt AMPure magnetic beads (Beckman Coulter). Subsequently, qPCR was performed (StepOne, Applied Biosystems) using ChIP and input DNA amplifying different regions around the transcriptional start site of *Acod1* (*Irg1*). Enrichment of TFEB binding was calculated as ChIP–DNA relative to input-DNA PCR signal for each primer pair and normalized to a negative control region (non-accessible heterochromatin region).

### Lysosomal mass measurements

BMDMs were incubated with 75 nM LysoTracker Red for 30 min at 37 °C in BMDM medium. Cells were washed three times with prewarmed BMDM culture medium, gathered with 20 mM EDTA and incubated for 15 min on ice with Live Dead Fixable Viability eFluor 780 (1:1,000) in PBS. Samples were measured on the BD LSR Fortessa cell analyser (BD Biosciences). Data were analysed and graphs generated in FlowJo v.10.

### Real-time qPCR

BMDMs were gathered in 250 µl TriReagent per well and RNA was isolated by phenol-chloroform extraction. cDNA synthesis was performed with the QuantiTect Reverse Transcription Kit according to the manufacturer’s instructions. As template, 200 ng of RNA were used. The reverse transcription reaction was performed for 30 min at 42 °C. Measurement of *Irg1*, *Tfeb*, *β-actin* and *β*_*2*_*-microglobulin* mRNA expression was carried out in a 96-well plate using the Thermo Scientific ABsolute Blue QPCR SYBR Green Low ROX Mix according to the manufacturer’s instructions using 1 µl of cDNA and 22 ng of the respective primers. Samples were measured in the 7500 Fast Real-Time PCR System (Applied Bioscience) and analysed via StepOne Software (AB, v.2.0). To quantify relative *Irg1* mRNA expression, *Irg1* mRNA levels were normalized to the expression of the housekeeping genes *β-actin* and *β*_*2*_*-microglobulin*. Relative mRNA expression values were calculated using the 2(-ΔΔCT) method and normalized to unstimulated control samples. For Fig. 3g and Extended Fig. 3d (2-DG treatment), *Irg1* expression levels were normalized to *Tfeb* expression levels per sample because the genotype of the cells or the treatment affected *Tfeb* expression levels.

### Seahorse flux analysis

Extracellular acidification rate and oxygen consumption rate were determined with a Seahorse Flux Analyser XF96 (Agilent Technologies) from GFP- or TFEB-GFP-expressing BMDMs. Seahorse XF base medium was supplemented with 25 mM glucose, 2 mM glutamine, 1 mM sodium pyruvate and 1% FCS. Seahorse measurements were normalized to protein content, determined with the Pierce BCA Protein Assay Kit or cell numbers by using in situ Hoechst staining of nuclei. Nuclear stainings were acquired with the BioTek Cytation 1/5. Nuclei counting was performed with the Seahorse XF and Cell Counting Software and the Wave v.2.6 Software (Agilent Technologies). Oxygen consumption rate data were calculated as area under the curve and values were plotted using GraphPad Prism v.8.2.1.

### Metabolic tracing

Metabolic tracing with ^13^C -glucose, ^13^C-glutamine and ^13^C-palmitate was performed with gas chromatography coupled to tandem mass spectrometry (GC–MS/MS). For glucose and glutamine tracing, BMDMs were incubated for 6 h in glucose- or glutamine-free BMDM medium supplemented with 11 mM ^13^C-glucose or 4 mM ^13^C -glutamine, respectively. For palmitate tracing, full BMDM medium containing 20 µM BSA-conjugated ^13^C-palmitic acid was used, as BMDMs were dying in lipid-deprived medium. To extract metabolites, BMDMs were washed once with ice-cold 0.9% NaCl in MilliQ-H_2_O, shock frozen in an ethanol-dry ice bath and collected with a cell lifter in ice-cold 80% methanol containing 1 µg ml^−1^ norvaline and 1 µg ml^−1^ adipic acid (internal standards). Cell debris was removed by centrifugation for 5 min at 20,000*g* and 4 °C. Methanol supernatants were collected and dried in a Genevac EZ-2 (SP Scientific). Metabolites were resuspended in 10 µl D27/methoxyamine mix (10 mg ml^−1^ methoxyamine hydrochloride, 0.2 µg ml^−1^ myristic-D_27_ acid in pyridine) for 1 h at 30 °C. Then 7.5 µl of the mix were derivatized with 15 µl of *N*-(*tert*-butyldimethylsilyl)-*N*-methyl-trifluoroacetamid, with 1% *tert*-butyldimethyl-chlorosilane (375934 Sigma Aldrich) for 60 min at 80 °C. Isotopomer distributions were measured using a DB5-MS GC column in a 7890 GC system (Agilent Technologies) combined with a 5977 MS system (Agilent Technologies). Data from tracing experiments are presented as ^13^C-labelled metabolite fractions of total respective metabolite level.

### Intracellular itaconate measurements

Polar metabolome quantifications were performed with LC–MS. BMDMs were stimulated as indicated. For metabolite extraction, cells were washed once with ice-cold 3% glycerol in MilliQ-H_2_O, followed by 5 min incubation on ice in prechilled 80% methanol. Methanol supernatants were collected and cell debris was removed by centrifugation for 5 min at 15,000*g* and 4 °C. Metabolite solutions were dried in a Genevac EZ-2 (SP Scientific) and subsequently resuspended in 15 µl of 90% acetonitrile containing ^13^C-yeast-standard (ISOtopic Solutions) as loading control. Suspensions were centrifuged for 10 min at 3,300*g* and 4 °C and 10 µl of each sample were transferred to a fresh container and used for mass spectrometry. Targeted metabolite quantification by LC–MS was carried out using an Agilent 1290 Infinity II UHPLC in line with an Agilent 6495 triple quadrupole–MS operating in MRM mode. MRM settings were optimized separately for all compounds using pure standards. LC separation was on a Phenomenex Luna propylamine column (50 × 2 mm, 3-μm particles), with, a solvent gradient of 100% buffer B (5 mM ammonium carbonate in 90% acetonitrile) to 90% buffer A (10 mM NH_4_ in water). Flow rate was from 1,000 to 750 µl min^−1^. Autosampler temperature was 5 °C and injection volume 2 µl. Values represent the area of the metabolite peaks from mass spectrometry as arbitrary units.

### Western blotting

BMDMs were collected in ice-cold PBS with a cell lifter and pelleted by centrifugation for 5 min at 500*g* and 4 °C. Cell pellets were lysed for 15 min on ice with lysis buffer (50 mM Tris, 150 mM NaCl, 0.1% Triton X-100, pH 7.4) with 1× Halt Protease Inhibitor Cocktail and 1× Phosphatase Inhibitor Cocktail and sheared with a 26 G insulin syringe. Cell debris was removed by centrifugation at 16,000*g* and 4 °C for 15 min. Then 25 to 35 µg of total protein was loaded on a 10 or 12% polyacrylamide gels. Protein transfer to a polyvinyl difluoride-membrane (Merck Millipore) was performed in a semidry blotting chamber for 90 min at 10 V. Membranes were blocked for 1 h in 5% milk in tris-buffered saline (TBS) with 0.1% Tween (TBST) at room temperature. Incubation with primary antibodies was performed overnight at 4 °C in buffers suggested for the specific antibody or in TBST containing 2% BSA. Incubation with secondary antibodies was performed for 1 h at room temperature in 5% milk in TBST. For signal detection, Amersham ECL Prime Western Blotting Detection Reagent was used and signals were acquired with the ChemiDoc Touch Gel Imaging System (Bio-Rad). Images were prepared for publication with the Image Lab v.5.2 TM Touch Software (Bio-Rad, v.1.0.0.15).

### Immunofluorescence

BMDMs were plated in tissue culture treated 24-well plates containing fibronectin-coated 12 mm glass coverslips. To visualize TFEB, HSP60 or *Salmonella*, BMDMs were fixed for 15 min at room temperature in 4% paraformaldehyde, prewarmed to 37 °C, followed by permeabilization in 0.2% Triton X-100 in PBS. To visualize endogenous Irg1, BMDMs were fixed and permeabilized in ice-cold 100% methanol for 15 min at −20 °C. In both cases, unspecific binding sites were blocked afterwards for 1 h at room temperature in blocking buffer (0.1% Tween20, 5% FCS in PBS). Cells were incubated at 4 °C for 16 h with primary antibodies in blocking buffer. Secondary antibody stainings were performed in blocking buffer for 1 h at room temperature. BMDMs were mounted in Fluoromount-G supplemented with or without 4,6-diamidino-2-phenylindole (DAPI).

### Confocal microscopy and image processing

Z-stacks were acquired with an inverted LSM880 or LSM780 Zeiss confocal microscope and ZEN software black edition (Carl Zeiss Microscopy, v.2.6). Brightness and contrast were adjusted and images prepared using Fiji ImageJ^69,70^. 3D-rendered images of mitochondria-pathogen interactions were generated using the surface tool of Imaris v.9.5.1 (Bitplane). For better visualization of differences in expression levels, Irg1 and *Salmonella*-mCherry fluorescent signals in Fig. 3b and Extended Fig. 4j were pseudocolored in ImageJ using the look-up table ‘red hot’.

### Image analysis

To quantify nuclear TFEB-signals 3D nuclear masks generated from DAPI signal were generated, using the Imaris v.9.4.1 (Bitplane) surface tool (smoothing 0.51, signal intensities were set manually for each image). To quantify cellular TFEB levels in TFEB-GFP- or GFP-expressing BMDMs, cellular masks were generated on the basis of ectopically expressed GFP signals, as described for nuclear TFEB levels.

Mitochondria-pathogen interactions were determined from single slice images using the Pearson’s Coefficient ImageJ Jacob colocalization software tool (https://imagej.nih.gov/ij/plugins/track/jacop.html)^69,70^. Colocalized pixels were identified in individual slices using the ImageJ colocalization tool (channel cut-off 50)^69,70^.

To determine the bacterial load of individual macrophages by imaging (Extended Data Figure 5f,g), maximum intensity projections of images taken from *Salmonella*-mCherry infected BMDMs were transformed to binary images and signal per cell were measured using the ImageJ analysis tool^69,70^. Measured signals were presented as frequency distributions in Extended Data Fig. 5g. Data were normalized for bacterial signals in TFEBa relative to vehicle-treated BMDMs for each independent experiment. The proliferating *Salmonella* subpopulation was determined on the basis of signal representing >15 bacteria per cell. Signals containing the growing *Salmonella* subpopulation (from bins 10–14) were summarized for vehicle and TFEBa-treated BMDMs for each genotype and depicted as ratio TFEBa/vehicle-treated in Fig. 4k.

### *Salmonella* infection of BMDMs

*S. enterica* serovar Typhimurium strain SL1344, harbouring the pFCcGi plasmid, was cultured for 16 h at 37 °C in a minimum MgMES medium (170 mM MES, 5 mM KCl, 7.5 mM (NH_4_)_2_SO, 0.5 mM K_2_SO_4_, 1 mM KH_2_PO_4_, 8 µM MgCl_2_, 38 M glycerol, 0.1% casamino acid, pH 5.8, 100 µg ml^−1^ ampicillin) supplemented with with 0.2% (w/v) l-arabinose and 100 µg ml^−1^ carbenicillin. Before infection of BMDMs, bacteria were opsonized for 20 min with 10% mouse serum in BMDM infection medium. BMDMs were pretreated or not with TFEBa for 3 h before opsonized living or heat-killed *Salmonella* were added. BMDMs were infected at a multiplicity of infection (MOI) of 5 for all experiments and incubated for 18.5 h postinfection. Host–bacteria interactions were synchronized by centrifugation for 10 min at 300*g* and room temperature. After 30 min, extracellular bacteria were killed by addition of gentamycin (100 µg ml^−1^) containing BMDM infection medium. After 30 min, gentamycin concentration was reduced to 10 µg ml^−1^, TFEBa or itaconate were added to BMDMs and cells were collected or incubated for further 18 h.

### Analysis of *Salmonella* subpopulations by flow cytometry

To determine intracellular *Salmonella* subpopulations (growing, non-growing, host-killed) infected BMDMs were washed once with cold PBS and then collected on ice with a cell lifter in PBS. Per condition, three technical replicates were pooled. To assess *Salmonella* subpopulations on the basis of GFP and mCherry signals, samples were measured on a BD FACSAria III cell sorter with FACSDiva (BD, v.8.0.1). Control gates were set on the basis of there being uninfected or 30-min infected BMDMs (Extended Data Fig. 4d).

### Plating assay to assess intra-macrophage bacterial survival rates

BMDMs infected for 18.5 h were washed once with cold PBS and immediately lysed in 1 ml of PBS with 0.1% Triton X-100. Serial dilutions (1:10, 1:100, 1:1,000) were plated on Luria-Bertani (LB) agar plates containing 100 µg ml^−1^ ampicillin and incubated at 37 °C for 16 h to allow *Salmonella* regrowth. CFUs were counted manually.

### Luciferase assays

NanoLuc-ITA-*Salmonella*-infected BMDMs were lysed 18.5 h post-infection in Passive Lysis Buffer (E1941, Promega) and luciferase activity was determined using the Nano-Glo-Luciferase Assay System (N1110, Promega) according to the manufacturer’s instructions and a Centro LB 963 Microplate Luminometer (Berthold). In parallel, CFUs were determined and luminescence values were normalized to the ratio of CFUs between TFEBa-treated and control BMDMs. For *Irg1*-promotor luciferase measurements, mouse embryonic fibroblasts (ATCC CRL-2907) were cotransfected with the indicated *Irg1*-promoter-firefly luciferase constructs and GFP as control or the indicated TFEB-GFP constructs. Then 24 h after transfection, cells were treated with medium or hk *Sm*T (10^5^ particles per ml) for 3 h and luciferase expression was measured using the Glo-Luciferase Kit (Promega) according to the manufacturer’s instructions on a Centro LB 963 Microplate Luminometer (Berthold). Luciferase-expression levels were quantified as fold increase relative to *Irg1*-promoter-luciferase/GFP-coexpressing control cells.

### Bacterial SPI-2 expression measurements

BMDMs were infected with *Salmonella* Typhimurium strain SL1344 carrying the P_*ssaG::gfp*_ plasmid^71^, encoding a GFP-reporter gene under the control of the bona fide SPI-2 promotor of the *ssaG* gene. Bacteria were grown overnight in LB medium containing 50 µg ml^−1^ chloramphenicol. Opsonization and BMDM infection were performed as described in the *Salmonella* infection assay. To assess SPI-2 GFP-reporter expression, BMDMs infected for 18.5 h were fixed at room temperature for 15 min in 4% paraformaldehyde before being measured on a BD LSR Fortessa cell analyser (BD Biosciences), with FACSDiva (BD, v.8.0.1). FACS data were analysed with FlowJo v.10 software.

### In vitro *Salmonella* survival assay

*Salmonella* was grown in 50 ml of LB medium to an optical density (OD_600_) of 2. Subsequently, 2.5 ml of bacterial suspension were collected, spun down and bacteria were resuspended in 5 ml of either LB medium (100 µg ml^−1^ ampicillin) or minimal medium (170 mM MES, 5 mM KCl, 7.5 mM (NH_4_)_2_SO, 0.5 mM K_2_SO_4_, 1 mM KH_2_PO_4_, 8 µM MgCl_2_, 38 mM glycerol, 0.1% casamino acid, pH 5.8, 100 µg ml^−1^ ampicillin). Bacterial cultures were incubated with TFEBa (5 mM) and bacterial growth/survival were inferred from CFUs.

### Quantification and statistical analysis

#### (Statistical analysis and data representation)

Graphs were generated and statistical analysis was carried out with GraphPad Prism v.8.2.1. To determine statistical significance, different tests were used as indicated in the figure legends. The number of experimental repeats is indicated in the figure legends. Proportional Venn diagrams for overlapping genes were generated with BioVenn^72^. Statistical significance of the overlap between the two groups of genes was calculated with a hypergeometric statistical test. Heatmaps were generated with Morpheus (Broad Institute) and schematics and figures with Adobe Illustrator CS5 (Adobe). Gene tracks of the *Irg1* locus were generated with the Integrative Genomics Viewer (IGV)^73^.

### Materials availability

No new, unique reagents, plasmids or mice were generated in this study. A Material Transfer Agreement exists for the use of *Tfeb*^*flf/fl*^ mice. These mice can only be shared via A.Ballabio.

### Reporting summary

Further information on research design is available in the Nature Research Reporting Summary linked to this article.

### Supplementary information

Supplementary Information Supplementary Tables 1 and 2 and Fig. 1.

Reporting Summary

### Source data

Source Data Fig. 1 Statistical source data.

Source Data Fig. 2 Statistical source data.

Source Data Fig. 3 Statistical source data.

Source Data Fig. 4 Statistical source data.

Source Data Extended Data Fig. 1 Statistical source data.

Source Data Extended Data Fig. 2 Unprocessed western blot.

Source Data Extended Data Fig. 2 Statistical source data.

Source Data Extended Data Fig. 3 Statistical source data.

Source Data Extended Data Fig. 4 Statistical source data.

Source Data Extended Data Fig. 5 Statistical source data.

Source Data Extended Data Fig. 5 Unprocessed western blot.

## Data Availability

RNA- and ATAC-seq data generated in this study have been deposited at Sequence Read Archive with the accession code PRJNA647627. The authors declare that all other data supporting the findings of this study are available within the paper and supplementary information files. Source data are provided with this paper.

## Extended data

are available for this paper at 10.1038/s42255-022-00605-w.
