# Supplementary material for: TFEB induces mitochondrial itaconate synthesis to suppress bacterial growth in macrophages
Source: Nat Metab. 2022 Jul 21;4(7):856–66. doi: 10.1038/s42255-022-00605-w (PMC9314259; doi:10.1038/s42255-022-00605-w)
Supplement: Supplementary file 1 — Supplementary Tables 1 and 2 and Fig. 1. [file 42255_2022_605_MOESM1_ESM.pdf]

nature portfolio

https://doi.org/10.1038/s42255-022-00605-w

Supplementary information

TFEB induces mitochondrial itaconate synthesis to suppress bacterial growth in macrophages

In the format provided by the authors and unedited

Supplementary Information

Motif	Transcription Factor	p-value
TCGTAGCACT	Zfp691	1e-4
CATTACCGTA	Homeobox protein Nkx-6.1	1e-4
GTGACTGTGTAT	FOXH1	1e-4
AAAGGCACACGT	Mlxip	1e-4
CACTAAAGGCCC	Homeobox protein Nkx-2.1	1e-4
AGCAACTACTCC	Hmx1	1e-4
ATCCACTCCTGA	ZNF354C	1e-4
GCCAGTTACCCT	Spz1	1e-4
GAGCAACATGAT	Homeobox protein Six1	1e-4
GCTCAAGGTATC	RAR:RXR (NR)	1e-4
GGCGCTTGCCTA	at_AC_acceptor	1e-4
TCCTGGCTTGAT	SREBF2	1e-4
TCCTTTCTCAAT	Irf3	1e-4
CAGTTGGTCTTT	Mybl1	1e-4
TGCTAGCTATCT	GATA	1e-4
GTTTGATCACCT	Cux1	1e-4
TCTACAAGTGAT	Zbtb3	1e-4
ACAAACCACAAT	RUNX	1e-4
CCTCCCACCTCG	TBX15	1e-4
CCCTTCACTGTT	PRDM1	1e-4
ACCCGTCACCTC	Eomes-2	1e-4
GTTGTCATGTGC	TFEB	1e-4
TACCAGCTTGCA	Tcf21	1e-4
ATTTTCACATCG	Eomes-1	1e-4
GTGAGTTACAAT	Homeobox protein Pknox1	1e-4
GCACTATCTACT	Mnx1	1e-4
CGGTCTAGCC	SMAD3	1e-4
CACGTCCACT	HIF-1A	1e-3
TACTTGAAAC	Nkx-2.2	1e-3
GTATAAAGGC	TATA-Box	1e-3
CCTAAACTGC	BMYB (HTH)	1e-3
ATGTGCTTAC	NRL	1e-3
GACACAACTC	KLF10	1e-3

Supplementary Table 1. Potential Irg1 regulators upon TFEB activation.

List of identified transcription factor binding motifs in significantly opened chromatin regions of the *Irg1* locus from ATAC-seq analysis (shown in Figure 4h) in TFEB-GFP- versus GFP-expressing BMDMs. Identified motifs are shown in column 1. Column 2 indicates the respective transcription factor. Column 3 represents the significance of enrichment of the binding motifs in the gained *Irg1* peaks calculated via the HOMER motif analysis software.

Supplementary Table 2
Oligonucleotide sequences

**Cloning primers**	
Irg1-forw	TTCAAGCTTGCCACCATGATGCTCAAGTCTGTCACAGAG
Irg1-rev	GGCCCGCGGTACCTTGATATTGGTAAACCTGGGCAACG
BFP-forw	CCACCGGTCGCCACCATGAGCGAGCTGATTAAGGAG
BFP-rev	TGTGCTGGCGGCCGCTCAATTAAGCTTGTGCCCCAG
**RT-qPCR primers**	
Irg1-forw	GCAACATGATGCTCAAGTCTG
Irg1-rev	TGCTCCTCCGAATGATACCA
Tfeb-forw	TGATCCCCAAGGCCAATGAC
Tfeb-forw	TCCCTGGACTTTTGCAGGTC
b2m fw	CTGCTACGTAACACAGTTCCACCC
b2m rev	CATGATGCTTGATCACATGTCTCG
Actin fw	TCGTTGCCGGTCCACACCCG
Actin rev	CTTTGCACATGCCGGAGCCGT
**ChIP-qPCR primer**	
-800bp_FW	GGCCACCATTCCTCACAAAT
-800bp_RV	ACGAGTAAGTGAGCATGGGT
-200bp_FW	GTCTTCAAGAGTGGGATGCC
-200bp_RV	ATAATTACAGAGGGATGGCCTG
negative-control_FW	TGCTCCACAGTGTCCATGTACA
negative-control_RV	AGCAATTTCATGGGTGAGAGAAG

Supplementary Figure

Flow cytometry gating strategy

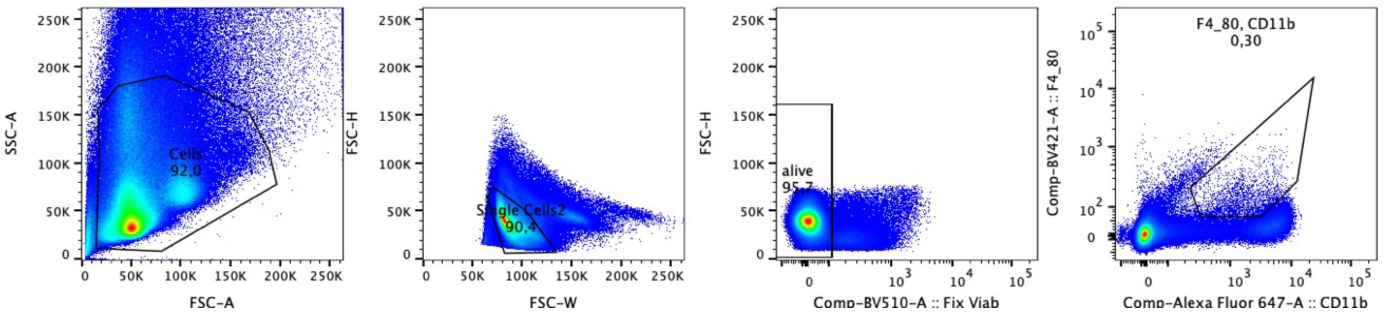

Supplementary Figure 1: Flow cytometry gating strategy to identify splenic macrophages.
Relvant for Figure 4d, g and Extended Data Figure 4 c,h. From splenic cells suspensions (SSC-A to FSC-A), doublets (FSC-W to FCH-H) and dead cells (viable to FSC-H) were excluded. Macrophages were identified as double positive F4/80+ and CD11b+ population.
