# Supplementary material for: TFEB induces mitochondrial itaconate synthesis to suppress bacterial growth in macrophages
Source: Nat Metab. 2022 Jul 21;4(7):856–66. doi: 10.1038/s42255-022-00605-w (PMC9314259; doi:10.1038/s42255-022-00605-w)
Supplement: Supplementary file 2 — Reporting Summary [file 42255_2022_605_MOESM2_ESM.pdf]

nature portfolio

Corresponding author(s): Angelika Rambold

Last updated by author(s): June 5th 2022

Reporting Summary

Nature Portfolio wishes to improve the reproducibility of the work that we publish. This form provides structure for consistency and transparency in reporting. For further information on Nature Portfolio policies, see our Editorial Policies and the Editorial Policy Checklist.

Statistics

For all statistical analyses, confirm that the following items are present in the figure legend, table legend, main text, or Methods section.

n/a	Confirmed	
[ ]	[x]	The exact sample size (n) for each experimental group/condition, given as a discrete number and unit of measurement
[ ]	[x]	A statement on whether measurements were taken from distinct samples or whether the same sample was measured repeatedly
[ ]	[x]	The statistical test(s) used AND whether they are one- or two-sided<br>*Only common tests should be described solely by name; describe more complex techniques in the Methods section.*
[x]	[ ]	A description of all covariates tested
[ ]	[x]	A description of any assumptions or corrections, such as tests of normality and adjustment for multiple comparisons
[ ]	[x]	A full description of the statistical parameters including central tendency (e.g. means) or other basic estimates (e.g. regression coefficient) AND variation (e.g. standard deviation) or associated estimates of uncertainty (e.g. confidence intervals)
[ ]	[x]	For null hypothesis testing, the test statistic (e.g. F, t, r) with confidence intervals, effect sizes, degrees of freedom and P value noted<br>*Give P values as exact values whenever suitable.*
[x]	[ ]	For Bayesian analysis, information on the choice of priors and Markov chain Monte Carlo settings
[x]	[ ]	For hierarchical and complex designs, identification of the appropriate level for tests and full reporting of outcomes
[ ]	[x]	Estimates of effect sizes (e.g. Cohen's d, Pearson's r), indicating how they were calculated

Our web collection on statistics for biologists contains articles on many of the points above.

Software and code

Policy information about availability of computer code

Data collection	For imaging data collection: Zen (Carl Zeiss Microscopy, v2.6)<br>For FACS data collection: FACSDiva (BD, v8.0.1), BD FACSAria III cell sorter, LSRFortessa cell analyzer (BD Biosciences)<br>For qPCR data collection: StepOne Software (AB, v2.0).<br>For Western Blot data collection: Image Lab 5.2 TM Touch Software (Bio-Rad, v1.0.0.15)<br>For RNa-seq collection: HiSeq 3000 (Illumina), PANTHER classification system version 13<br>For ATAC seq collection: HiSeq3000 (Illumina)
Data analysis	For Imaging data analysis: Imaris (Bitplane, v9.5.1, v9.4.1), Fiji (v2.0.0-rc-43/1.51a)<br>For FACS data analysis: FlowJo (BD, v10.5.3).<br>For qPCR data analysis: Excel (Microsoft, v14.7.3)<br>For statistical data analysis and generation of graphs: Graphpad Prism (v8.2.1)<br>For RNA-seq analysis:<br>featureCounts 5 (https://doi.org/10.1093/ bioinformatics/btt656). DESeq2 algorithm 6 (https://doi.org/10.1186/s13059-014-0550-8), R, 3.6.0 (Lucent Technologies), PANTHER classification system Version 13 (http://www.pantherdb.org) using the Gene List analysis (Statistical overrepresentation test, Annotation Data Set: PANTHER GO- Slim Biological Process, Reference List: Default Mus musculus genes<br>For ATAC seq analysis: BEDTools 12 (version v2.25.0), DeepTools 13 v3.5.0, HOMER 14 v4.1.0 software with "findMotifsGenome.pl" command, FIMO 15 from the MEME suite v5.3.3.<br>For statistical data analysis and generation of graphs: Graphpad Prism (v8.2.1)

For manuscripts utilizing custom algorithms or software that are central to the research but not yet described in published literature, software must be made available to editors and reviewers. We strongly encourage code deposition in a community repository (e.g. GitHub). See the Nature Portfolio guidelines for submitting code & software for further information.

Data

Policy information about availability of data

All manuscripts must include a data availability statement. This statement should provide the following information, where applicable:

- Accession codes, unique identifiers, or web links for publicly available datasets
- A description of any restrictions on data availability
- For clinical datasets or third party data, please ensure that the statement adheres to our policy

RNA sequencing and ATAC sequencing data generated in this study have been deposited to SRA. SRA accession code: PRJNA647627. The authors declare that all other data supporting the findings of this study are available within the paper (and its source data and supplementary information files).

Field-specific reporting

Please select the one below that is the best fit for your research. If you are not sure, read the appropriate sections before making your selection.

[x] Life sciences   [ ] Behavioural & social sciences   [ ] Ecological, evolutionary & environmental sciences

For a reference copy of the document with all sections, see nature.com/documents/nr-reporting-summary-flat.pdf

Life sciences study design

All studies must disclose on these points even when the disclosure is negative.

Sample size	Sample sizes were determined on the basis of previous experience in previous experiments. For infections studies 3-5 independent experiments with BMDMs or mice (based on Saliba et al., 2016 Nat. Microbiol, Chen et al., Science 2019), for vacuolar itaconate measurements of 3 independent experiments (based on Chen et la., Science 2019) were sufficient to detect meaningful biological differences with good reproducibility. For metabolomics, qPCR measurements, imaging experiments we used at least 3-4 independent experiments. This number of independent experiments is a standard sample size to accurately detect differences in cell biology experiments (such as Michelucci et a., PNAS 2013, Napolitano et al., Nature 2020. https://doi.org/10.1038/s41586-020-2444-0). Western Blots and images are shown as representative images. However, they were reproduced in a least three independent experiments or with at least 3 biologically independent samples.
Data exclusions	For metabolomics analysis: data sets were excluded when control cells (e.g. LPS/IFNg-treatment) failed to upregulate itaconate.<br>For image analysis: Cells were excluded when poor staining qualities did not allow image acquisition or analysis. Also cells that overlapped with one another were excluded.<br>For FACS analysis: In cases where control conditions did not show growing Salmonella populations, or where bacterial loads of splenic macrophages did not exceed levels found in non-infected control animals, these data sets were not used for further analysis.
Replication	Reproducibility of the experimental findings was verified using biological replicates and independent experiments. The numbers are indicated in the respective figures (individual data points). In addition, the majority of our Salmonella-infected studies were performed in two labs (Alexander Westermann and Rambold lab), and thus have been replicated by independent laboratories.
Randomization	For experiments performed on cells, mice were randomly assigned. Animals ordered from the breeding facility were assigned to cages by the facility staff without knowledge of the experimental setup for which the mice were intended. The cages were randomly assigned to treatments.Experimental groups are defined by the genotype or treatments.
Blinding	Experimentalists were blinded regarding BMDMs genotype. Mouse numbers were used as identifiers. Image acquisition and FACS analysis and metabolic measurements and analysis were performed blinded.

Behavioural & social sciences study design

All studies must disclose on these points even when the disclosure is negative.

Study description	*Briefly describe the study type including whether data are quantitative, qualitative, or mixed-methods (e.g. qualitative cross-sectional, quantitative experimental, mixed-methods case study).*
Research sample	*State the research sample (e.g. Harvard university undergraduates, villagers in rural India) and provide relevant demographic information (e.g. age, sex) and indicate whether the sample is representative. Provide a rationale for the study sample chosen. For studies involving existing datasets, please describe the dataset and source.*
Sampling strategy	*Describe the sampling procedure (e.g. random, snowball, stratified, convenience). Describe the statistical methods that were used to predetermine sample size OR if no sample-size calculation was performed, describe how sample sizes were chosen and provide a rationale for why these sample sizes are sufficient. For qualitative data, please indicate whether data saturation was considered, and what criteria were used to decide that no further sampling was needed.*
Data collection	*Provide details about the data collection procedure, including the instruments or devices used to record the data (e.g. pen and paper, computer, eye tracker, video or audio equipment) whether anyone was present besides the participant(s) and the researcher, and*

	whether the researcher was blind to experimental condition and/or the study hypothesis during data collection.
Timing	*Indicate the start and stop dates of data collection. If there is a gap between collection periods, state the dates for each sample cohort.*
Data exclusions	*If no data were excluded from the analyses, state so OR if data were excluded, provide the exact number of exclusions and the rationale behind them, indicating whether exclusion criteria were pre-established.*
Non-participation	*State how many participants dropped out/declined participation and the reason(s) given OR provide response rate OR state that no participants dropped out/declined participation.*
Randomization	*If participants were not allocated into experimental groups, state so OR describe how participants were allocated to groups, and if allocation was not random, describe how covariates were controlled.*

Ecological, evolutionary & environmental sciences study design

All studies must disclose on these points even when the disclosure is negative.

Study description	*Briefly describe the study. For quantitative data include treatment factors and interactions, design structure (e.g. factorial, nested, hierarchical), nature and number of experimental units and replicates.*
Research sample	*Describe the research sample (e.g. a group of tagged Passer domesticus, all Stenocereus thurberi within Organ Pipe Cactus National Monument), and provide a rationale for the sample choice. When relevant, describe the organism taxa, source, sex, age range and any manipulations. State what population the sample is meant to represent when applicable. For studies involving existing datasets, describe the data and its source.*
Sampling strategy	*Note the sampling procedure. Describe the statistical methods that were used to predetermine sample size OR if no sample-size calculation was performed, describe how sample sizes were chosen and provide a rationale for why these sample sizes are sufficient.*
Data collection	*Describe the data collection procedure, including who recorded the data and how.*
Timing and spatial scale	*Indicate the start and stop dates of data collection, noting the frequency and periodicity of sampling and providing a rationale for these choices. If there is a gap between collection periods, state the dates for each sample cohort. Specify the spatial scale from which the data are taken*
Data exclusions	*If no data were excluded from the analyses, state so OR if data were excluded, describe the exclusions and the rationale behind them, indicating whether exclusion criteria were pre-established.*
Reproducibility	*Describe the measures taken to verify the reproducibility of experimental findings. For each experiment, note whether any attempts to repeat the experiment failed OR state that all attempts to repeat the experiment were successful.*
Randomization	*Describe how samples/organisms/participants were allocated into groups. If allocation was not random, describe how covariates were controlled. If this is not relevant to your study, explain why.*
Blinding	*Describe the extent of blinding used during data acquisition and analysis. If blinding was not possible, describe why OR explain why blinding was not relevant to your study.*

Did the study involve field work? ☐ Yes ☐ No

Field work, collection and transport

Field conditions	*Describe the study conditions for field work, providing relevant parameters (e.g. temperature, rainfall).*
Location	*State the location of the sampling or experiment, providing relevant parameters (e.g. latitude and longitude, elevation, water depth).*
Access & import/export	*Describe the efforts you have made to access habitats and to collect and import/export your samples in a responsible manner and in compliance with local, national and international laws, noting any permits that were obtained (give the name of the issuing authority, the date of issue, and any identifying information).*
Disturbance	*Describe any disturbance caused by the study and how it was minimized.*

Reporting for specific materials, systems and methods

We require information from authors about some types of materials, experimental systems and methods used in many studies. Here, indicate whether each material, system or method listed is relevant to your study. If you are not sure if a list item applies to your research, read the appropriate section before selecting a response.

Materials & experimental systems

n/a	Involved in the study	
☐	☒	Antibodies
☐	☒	Eukaryotic cell lines
☒	☐	Palaeontology and archaeology
☐	☒	Animals and other organisms
☒	☐	Human research participants
☒	☐	Clinical data
☒	☐	Dual use research of concern

Methods

n/a	Involved in the study	
☒	☐	ChIP-seq
☐	☒	Flow cytometry
☒	☐	MRI-based neuroimaging

Antibodies

Antibodies used

The following primary antibodies were used:
anti-IRG1 (IF 1:100; Abcam, ab222411),
anti-HSP60 (IF 1:1,000; CST, D6F1)
anti-TFEB (WB: 1:3,000; IF: 1:1,000; Bethyl Laboratories, A303-673A), anti-TFEB (ChIP, (D2O7D) Rabbit mAb #37785, Cell Signaling, 1:50).
anti-Salmonella Typhimurium control serum (IF 1:10,000 TS1624, Sifin;)
anti-Rab32 (WB 1:2000, LS C204248, LSBBio)
anti-actin (WB 1:500; SantaCruz, sc-47778).
anti-rabbit HRP-linked (WB 1:8,000; CST, 7074),
anti-goat HRP-linked (WB 1:8,000; ThermoFisher, 31402),
anti-rabbit Alexa Fluor 647-conjugated (IF, 1:500; ThermoFisher, A-21244),
anti-rabbit Cy3-conjugated (IF, 1:1,000; Jackson Immuno Research Laboratories, 111-165-144).

Validation

All commercial antibodies, validation on company websites:
https://www.abcam.com/irg1-antibody-epr22066-ab222411.html
https://www.thermofisher.com/antibody/product/HSP60-Antibody-clone-3G8-Monoclonal/MA5-15836
https://www.bethyl.com/product/A303-673A/TFEB+Antibody, https://www.cellsignal.com/products/primary-antibodies/tfeb-d2o7d-rabbit-mab/37785
https://www.sifin.de/produkte/bakteriologische-testreagenzien/salmonella-diagnostik/kontrollseren-fuer-die-salmonella-o-und-oh-testantigene/
https://www.lsbio.com/antibodies/rab32-antibody-c-terminus-if-immunofluorescence-wb-western-ls-c204248/212504
https://www.scbt.com/de/p/actin-antibody-i-19
https://www.thermofisher.https://www.thermofisher.com/antibody/product/Goat-anti-Rabbit-IgG-H-L-Cross-Adsorbed-Secondary-Antibody-Polyclonal/A-21244com/antibody/product/Rabbit-anti-Goat-IgG-H-L-Secondary-Antibody-Polyclonal/31402
https://www.jacksonimmuno.com/catalog/products/111-165-144

Eukaryotic cell lines

Policy information about cell lines

Cell line source(s)

immortalized mouse embryonic fibroblasts (MEFs) were sourced from ATCC (CRL-2907™), PlatE were sourced from R. Grosschedl (MPI-IE Freiburg), immortalized BMDMs were sourced from T. Lämmermann (MPI-IE, Freiburg).

Authentication

MEFs were authenticated by ATCC, no additional authentication was performed. PlatE cells were not authenticated. iBMDMs were generated from primary BMDMs, no additional authentication was performed.

Mycoplasma contamination

Cell lines are regularly checked for being negative for Mycoplasma infection.

Commonly misidentified lines
(See ICLAC register)

No commonly misidentified cell lines were used in this study.

Animals and other organisms

Policy information about studies involving animals; ARRIVE guidelines recommended for reporting animal research

Laboratory animals

C57BL/6 mice (RRID: IMSR_JAX:000664), Tfebfl/fl mice with Vav-iCre transgenicor Tfebfl/fl or Lyz2-cre mice, Irg1−/− mice (strain C57BL/6NJ-Acod1em1(IMPC)J/J), Irf1−/− mice (B6.129S2-Irf1tm1Mak/J) and Hps4−/− mice (B6.C3-Pde6brd1 Hps4le/J), Ifnar1−/− mice (B6.129s2-Ifnartm(Neo)Agt), Souris−/− mice (strain C57BL/6J-Lystbg-Btlr/Mmucd), Atg7fl/fl Vav-iCre mice. 8-25 weeks old male and female mice were used in this study. Mice were housed under controlled conditions, namely 20–21°C, 55–65% relative humidity, and 12:12 light-dark cycle. Food was available ad libitum for all animals. Mice were maintained, infected and euthanized under protocols approved by the animal care committee of the Regierungspräsidium Freiburg, Germany, in compliance with all relevant ethical regulations. Animals were euthanized by carbon dioxide asphyxiation followed by cervical dislocation, and bone marrow or spleens were harvested post mortem.

Wild animals	No wild animals were used for this study.
Field-collected samples	No field-collected samples were used for this study.
Ethics oversight	All mice were maintained in specific pathogen-free conditions and infected and euthanized under protocols approved by the animal care committee of the Regierungspräsidium Freiburg, Germany, in compliance with all relevant ethical regulations.

Note that full information on the approval of the study protocol must also be provided in the manuscript.

Flow Cytometry

Plots

Confirm that:

- [x] The axis labels state the marker and fluorochrome used (e.g. CD4-FITC).
- [x] The axis scales are clearly visible. Include numbers along axes only for bottom left plot of group (a 'group' is an analysis of identical markers).
- [x] All plots are contour plots with outliers or pseudocolor plots.
- [x] A numerical value for number of cells or percentage (with statistics) is provided.

Methodology

Sample preparation	To determine intracellular Salmonella subpopulations (growing, non-growing, host-killed), infected BMDMs were washed once with cold PBS and then harvested on ice with a cell lifter in PBS. Per condition, 3 technical replicates were pooled. To assess Salmonella subpopulations based on GFP and mCherry signals, samples were measured on a BD FACSAria III cell sorter. Spleens of infected mice were isolated, and cell suspensions were obtained by homogenizing the spleens using 70µm cell strainers. Erythrocyte lysis (ACK lysing buffer, Gibco A1049201) was performed and unspecific binding was blocked with anti-CD16/32 for 15 min before cells were stained for F4/80, Cd11b and live/dead in cold PBS for 1 h. Cells were fixed for 15 min using the Foxp3 transcription factor staining buffer set (eBioscience, 00-5523-00). Data was acquired on a LSR Fortessa (BD) and analyzed with FlowJo software (BD, version 10). During analysis, doublets were excluded.
Instrument	FACSAria III cell sorter (BD Biosciences), BD LSRFortessa (BD Biosciences)
Software	BD FACSDiva 8.0.1 for data collection, FlowJo (BD Biosciences, v10.5.3) for data analysis.
Cell population abundance	N/A
Gating strategy	The gating strategy is incorporated in the figure. The Salmonella-treated BMDM population was gated on single cells according to FSC-A/FSC-H. Salmonella sub-populations inside BMDMs were identified based on the relative GFP and mCherry signals. Uninfected and 30-minutes infected BMDMs were used as controls, as shown in Figure 4. Splenic cells from Salmonella-infected and non-infected control mice, were gated on single cells, according to FSC-A/FSC-H, on living cells based on stainings with a live/dead marker. Macrophage populations were identified by Cd11b+/F4/80+ stainings. Salmonella-mCherry-infected cells were identified in the macrophage population (Extended Data Figure 4g).

- [x] Tick this box to confirm that a figure exemplifying the gating strategy is provided in the Supplementary Information.
