# Supplementary material for: TFEB induces mitochondrial itaconate synthesis to suppress bacterial growth in macrophages
Source: Nat Metab. 2022 Jul 21;4(7):856–66. doi: 10.1038/s42255-022-00605-w (PMC9314259; doi:10.1038/s42255-022-00605-w)
Supplement: Supplementary file 8 — Unprocessed western blot. [file 42255_2022_605_MOESM8_ESM.pdf]

Source data Extended Fig. 2g
Unprocessed Western Blots

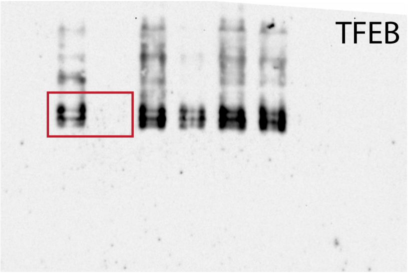

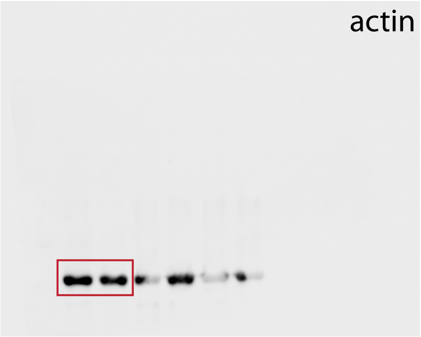

Red rectangles:
lanes used for
Extended Data
Figure 2g.
