# Supplementary material for: TFEB induces mitochondrial itaconate synthesis to suppress bacterial growth in macrophages
Source: Nat Metab. 2022 Jul 21;4(7):856–66. doi: 10.1038/s42255-022-00605-w (PMC9314259; doi:10.1038/s42255-022-00605-w)
Supplement: Supplementary file 13 — Unprocessed western blot. [file 42255_2022_605_MOESM13_ESM.pdf]

Source data to Extended Data 5b

Unprocessed Western Blots

Extended Data Figure 2b, left panel

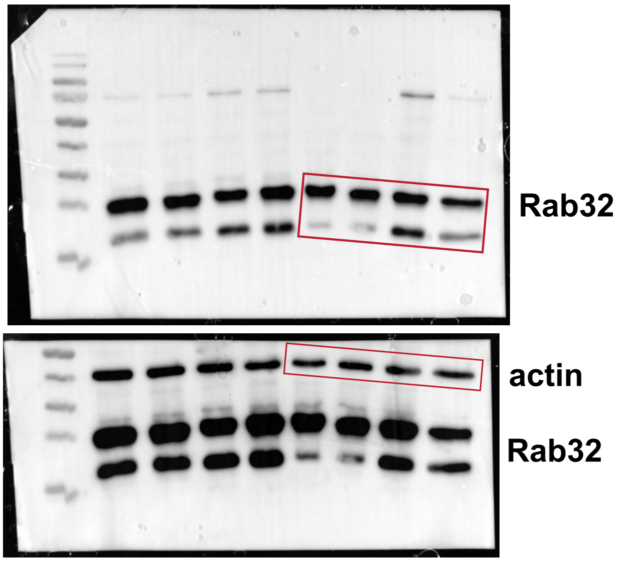

Red rectangles: lanes used for
Extended Data Figure 5b left panel.

Extended Data Figure 2b, right panel

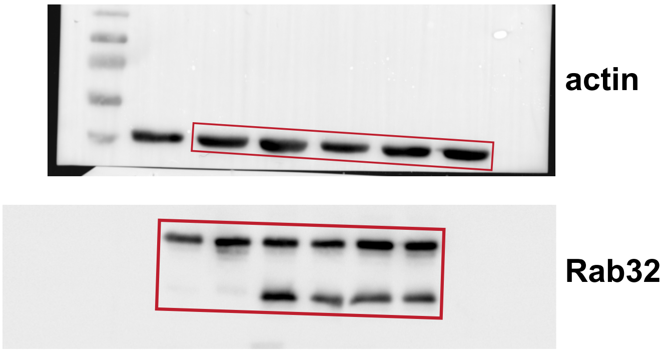

Red rectangles: lanes used for
Extended Data Figure 5b right panel.
